# Unraveling the Causal Relationship Between Blood Metabolites and Acne: A Metabolomic Mendelian Randomization Study

**DOI:** 10.1111/jocd.16763

**Published:** 2024-12-31

**Authors:** Min Li, Dan Dan Zhan, Li Li Fan, Yu Wang, Xiao Han Hu, Ming Zhang, Zhou Zhou

**Affiliations:** ^1^ Department of Dermatology Chinese People's Liberation Army Western Theater Command General Hospital Chengdu China

**Keywords:** acne, blood metabolites, causality, colocalization analysis, Mendelian randomization, metabolic pathways

## Abstract

**Background:**

Acne is a common skin disorder that may be linked to metabolic dysfunction. However, the causal impact of blood metabolites on acne has not been thoroughly investigated.

**Methods:**

We performed a metabolome‐wide Mendelian randomization (MR) analysis on 486 blood metabolites and acne using a genome‐wide association dataset. The study included preliminary inverse‐variance weighted (IVW) analysis, multivariable MR analysis, linkage disequilibrium score (LDSC) analysis, and colocalization analysis, along with reverse MR to address potential reverse causation.

**Results:**

Our analysis identified 12 metabolites significantly associated with acne. LDSC analysis revealed a genetic correlation between nonanoylcarnitine and acne. Colocalization analysis confirmed shared genetic variants, and metabolic pathway analysis implicated the arginine biosynthesis pathway and the selenocompound metabolism pathway in the development of acne.

**Conclusion:**

This study offers a comprehensive understanding of the causal relationships between plasma metabolites and acne. The findings provide insights into potential biomarkers and therapeutic targets for acne treatment, underscoring the need for further research.

## 1 Introduction

Acne vulgaris is a chronic inflammatory skin condition primarily affecting adolescents and young adults [1]. Despite its high prevalence and significant psychosocial impact, the pathogenesis of acne remains incompletely understood [2]. Metabolomic studies have identified altered metabolites in acne patients, suggesting their involvement in disease development [3, 4].

Mendelian randomization (MR) is a statistical method that uses genetic variants as instrumental variables (IVs) to determine causal relationships between exposures and outcomes [5, 6]. In acne research, MR has been employed to investigate potential causal associations between acne and various health outcomes [7]. However, no study has yet utilized a metabolomic MR approach to explore the causal relationship between a wide range of blood metabolites and acne.

This study aims to address this research gap by conducting a metabolomic MR analysis. We hypothesize a causal association between specific blood metabolites and acne, which could lead to new biochemical insights into acne prevention and treatment. Additionally, personalized therapeutic strategies based on individual metabolic profiles could be developed. Overall, this research aims to enhance our understanding of acne and improve its management.

## 2 Methods

**FIGURE 1:**
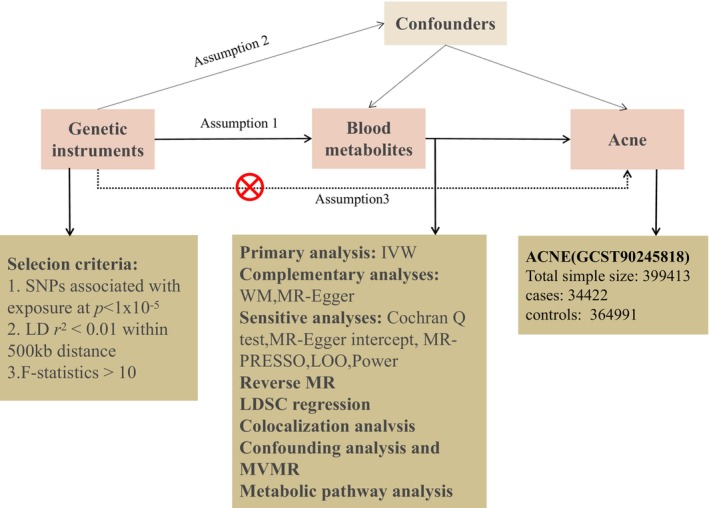
Overview of the Mendelian randomization (MR) analysis. Assumption (1) Strong correlation between genetic instruments and targeted exposures; Assumption (2) Independence of genetic variation from confounders related to the outcome; Assumption (3) Genetic instruments influence the outcome solely through the exposure. IVW, inverse‐variance weighted; LD, linkage disequilibrium; LDSC, linkage disequilibrium score; LOO analysis, leave‐one‐out analysis; MR‐PRESSO, MR‐Pleiotropy RESidual sum and outlier; MVMR, multivariable Mendelian randomization; SNPs, single nucleotide polymorphisms; WM, weighted median.

This study utilized an ethically approved, publicly accessible dataset that provided all the necessary data for the investigation. We thoroughly examined 486 serum metabolites to evaluate their association with acne risk, employing a rigorous MR design. To ensure scientific rigor in an MR study, three key assumptions must be satisfied: (1) a strong association between the genetic instruments and the exposures of interest, (2) independence of the genetic variants from confounding factors related to the outcome, and (3) the genetic instruments influence the outcome solely through the exposure. The main components of this study are illustrated in Figure 1.

### 2.1 GWAS Data Sources for 486 Blood Metabolites and Acne

Summary GWAS data for human serum metabolites were obtained from the Metabolomics GWAS Server (http://metabolomics.helmholtz‐muenchen.de/gwas/). This dataset is the most comprehensive available to date, encompassing blood metabolites identified by Shin et al. [8]. This study involved two major adult cohort studies: the TwinsUK cohort, which comprises 93% women with ages ranging from 17 to 85 years, all of whom are of British descent; and the KORA cohort, which includes German adults aged between 32 and 77 years with a balanced sex distribution. The study employed a cross‐sectional design and utilized liquid chromatography and gas chromatography coupled with tandem mass spectrometry to analyze blood metabolites. Rigorous quality control procedures were implemented to ensure the accuracy and reliability of the data. Their analysis included 529 metabolites across various categories, including amino acids, carbohydrates, coenzymes and vitamins, energy metabolites, lipids, nucleotides, peptides, and exogenous metabolites. The researchers ensured that the 486 metabolites ultimately used for genetic analysis were of high quality and reliability, suitable for subsequent statistical and genetic association studies. This extensive coverage facilitates a comprehensive understanding of the genetic regulation of metabolites. Several subsequent MR studies have utilized the GWAS data from this research [9, 10]. In the study by Shin et al., researchers conducted a genome‐wide association study (GWAS) on 7824 individuals of European descent to identify genetic loci influencing human blood metabolites. They assessed the association of over 2.1 million single nucleotide polymorphisms (SNPs) with 486 metabolites, including 309 known and 177 unknown metabolites, using linear regression models. Covariates such as age, sex, and batch effects were adjusted for in the analysis. To control the false positive rate from multiple testing, they employed Bonferroni correction and multivariate genetic analysis. Additionally, inverse variance meta‐analysis was utilized to combine data from two independent cohorts, thereby enhancing statistical power and reliability. The known metabolites are classified into eight categories based on the Kyoto Encyclopedia of Genes and Genomes (KEGG) database: cofactors and vitamins, energy, amino acids, carbohydrates, lipids, nucleotides, peptides, and xenobiotics (Table S1).

The GWAS data for acne used in this study were sourced from the GWAS Catalog, specifically the dataset with accession number GCST90245818. This dataset includes 399 413 subjects, with 34 422 acne cases identified through clinical assessments, ICD‐10 codes, or self‐diagnoses, and 364 991 controls, across three independent European‐ancestry cohorts [11].

### 2.2 Selection of IVs

The instrumental variables in our Mendelian randomization analysis were SNPs strongly associated with the trait of interest. These SNPs were selected based on their robust associations with the trait and minimal linkage disequilibrium with other loci, which allows us to infer causal relationships between the traits with greater confidence [12]. To identify suitable instrumental variables (IVs) for blood metabolites, we applied a rigorous set of criteria: SNPs with a minor allele frequency below 1% and significant missing data were excluded. Additionally, we used a significance threshold of *p* < 1×10^−5^, an LD value (*r*2) below 0.01 within a 500 kb region, and an F‐statistic of at least 10 [13]. SNPs that did not meet these criteria were excluded, and we retained those relevant to the exposure of interest while removing those associated with the outcome (*p* < 1 × 10^−5^). SNP harmonization was performed to resolve any inconsistencies in alleles between the exposure and outcome variables. To account for potential confounders, we carefully analyzed the IVs for metabolites using the Phenoscanner V2 platform. We examined the relationships between each SNP and known acne risk factors. SNPs that exhibited significant associations were excluded from further MR analysis to ensure result validity [14].

### 2.3 Primary Analysis and Sensitivity Analysis

The inverse‐variance weighted (IVW) method was utilized as the primary estimation approach due to its accuracy and validity assumptions [15]. The IVW method estimates causal effects by combining genetic associations with the exposure and outcome, weighting SNPs according to the inverse of their variances to achieve precise estimates [16]. Key parameters include the effect estimates and their standard errors, which are used to compute a weighted average causal effect. Supplementary analyses were conducted using the weighted median (WM) and MR‐Egger methods to refine our estimates under different assumptions [13, 17].

Sensitivity analysis included Cochran's Q test, the MR‐Egger intercept, Mendelian Randomization Pleiotropy RESidual Sum and Outlier (MR‐PRESSO), and Leave‐One‐Out (LOO) analysis [18, 19, 20]. Candidate metabolites involved in acne pathogenesis were identified based on consistency across multiple MR techniques, absence of pleiotropy or heterogeneity, and minimal impact from individual SNPs. The statistical power assessment was conducted using an online tool (https://shiny.cnsgenomics.com/mRnd/), calculating the statistical power based on the *r* ^2^ values of the IVs, case proportion, and odds ratios (OR) derived from the IVW analysis.

### 2.4 Reverse Direction MR Analysis

To evaluate the causal relationship between acne and blood metabolites, we performed a reverse direction MR analysis on metabolites that were identified as causally associated with acne in the forward MR analysis. We set a significance threshold of *p* < 5 × 10^−5^ and an LD *r* ^2^ value of less than 0.01 within a 500 kb range for the identification of IVs related to acne [21].

### 2.5 Multivariable MR Analysis

Multivariable Mendelian randomization (MVMR) analysis was employed to assess the causal relationships between various exposures and acne. By applying IVW methods [22], the MVMR analysis enabled us to estimate the independent effects of each metabolite on acne, accounting for variability in the outcomes.

### 2.6 Genetic Correlation Analysis

To account for potential confounding due to the coinheritance of exposure and outcome, we employed linkage disequilibrium score (LDSC) regression. This method uses chi‐squared statistics to estimate the coinheritance between two traits based on SNP data [23].

### 2.7 Colocalization Analysis

Colocalization analysis [24] was performed using the coloc R package to evaluate the potential effects of specific genomic loci on the relationships between identified metabolites and acne. This Bayesian approach examined five hypotheses, assigning posterior probabilities (H0, H1, H2, H3, and H4) to each, including the possibility of a shared causal variant. Associations with a posterior probability greater than 80% (PPH4) were deemed highly colocalized [25]. Default priors (p1 = 1 × 10^−4^, p2 = 1 × 10^−4^, and p12 = 1 × 10^−5^) were used in the analysis.

### 2.8 Metabolic Pathway Analysis

To explore the underlying biological mechanisms through which blood metabolites exert causal effects on acne, metabolic pathway analyses were performed using MetaboAnalyst 5.0 (https://www.metaboanalyst.ca/) [26].

## 3 Results

### 3.1 Selection of IVs

The number of selected instruments for each metabolite varied from 3 to 312, with a median of 13 instruments (Table S2). Notably, all F statistics exceeded the empirical threshold of 10, with the lowest F statistic being 17.1, confirming the robustness and validity of the SNPs utilized as instruments. We utilized Phenoscanner to conduct a phenotype query for metabolite SNPs, and the resulting outcomes have been recorded in Table S9. Notably, no phenotypes explicitly associated with the risk of acne were identified, thus suggesting that metabolite proxy SNPs remain unaffected by confounding factors related to acne.

### 3.2 Primary Analysis and Sensitivity Analysis

**FIGURE 2:**
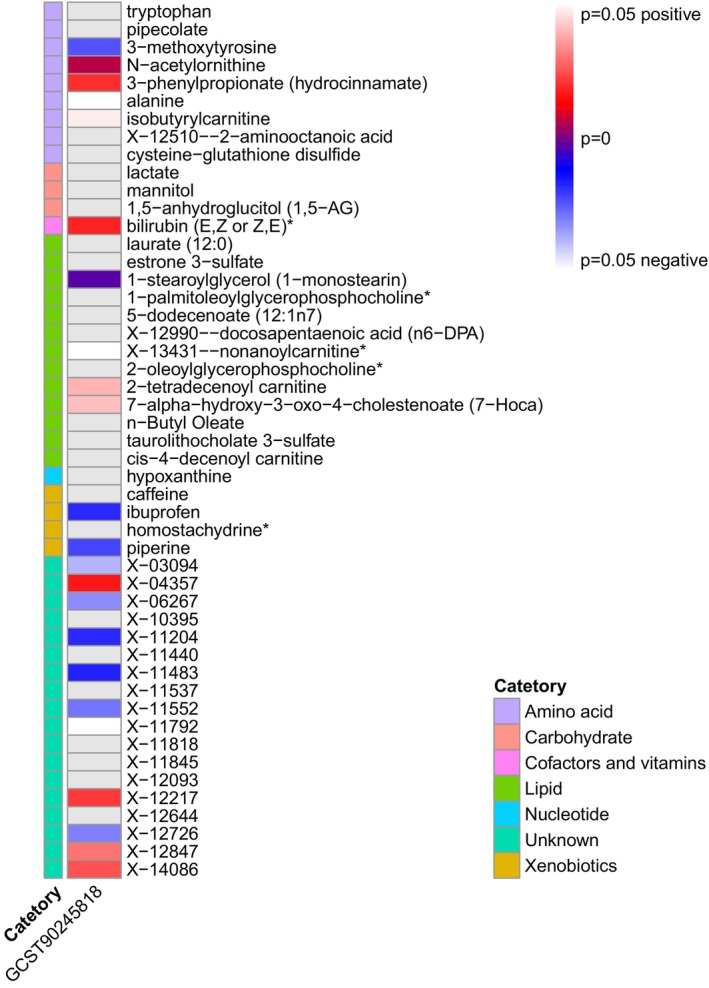
Heatmap showing potential causal metabolites (IVW: *p* < 0.05) linked to acne.

The IVW analysis was conducted to initially identify 30 candidate metabolites (Table S3). As shown in Figure 2, all known metabolites were classified into categories such as amino acids, carbohydrates, cofactors and vitamins, lipids, nucleotides, and xenobiotics.

Following this, complementary analyses using the WM and MR‐Egger methods were performed to screen for significance. Our primary goal was to establish statistical significance (*p* < 0.05) for the IVW estimates, while ensuring consistency in direction and magnitude across IVW, MR‐Egger, and WM (Figure S1).

After applying the MR‐PRESSO analysis to detect and exclude outlier SNPs, we effectively addressed SNP heterogeneity (Table S4). Subsequent assessments, including the Cochran Q test (*p* > 0.05) and MR‐Egger intercept test (*p* > 0.05), provided strong evidence against heterogeneity and pleiotropy (Table S5). LOO analysis results confirmed no bias in MR estimates due to individual SNPs (Figure S2). Finally, exposures with statistical power exceeding 80% were selected for further analysis (Table S6).

**FIGURE 3:**
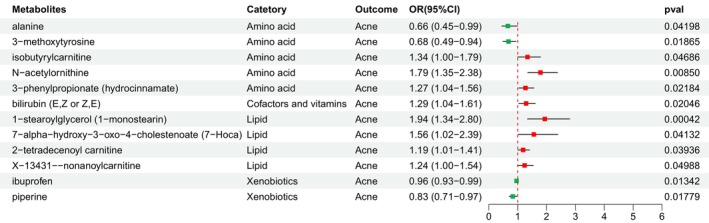
Forest plot illustrating the causal effect of metabolites on acne risk. CI, confidence interval; IVW, inverse‐variance weighted; OR, odds ratio.

Following the combined primary and sensitivity analyses, we identified 12 eligible known metabolites that met stringent screening criteria, while excluding 11 unknown metabolites (Table S7). These metabolites include 1‐stearoylglycerol (1‐monostearin) (OR, 1.94; 95% CI, 1.34–2.80; *p* = 0.0004), alanine (OR, 0.66; 95% CI, 0.45–0.99; *p* = 0.042), 3‐methoxytyrosine (OR, 0.68; 95% CI, 0.49–0.94; *p* = 0.0187), isobutyrylcarnitine (OR, 1.34; 95% CI, 1.00–1.79; *p* = 0.0469), ibuprofen (OR, 0.96; 95% CI, 0.93–0.99; *p* = 0.0134), *N*‐acetylornithine (OR, 1.79; 95% CI, 1.35–2.38; *p* = 0.0085), bilirubin (OR, 1.29; 95% CI, 1.04–1.61; *p* = 0.0205), 7‐alpha‐hydroxy‐3‐oxo‐4‐cholestenoate (7‐Hoca) (OR, 1.56; 95% CI, 1.02–2.39; *p* = 0.0413), 3‐phenylpropionate (hydrocinnamate) (OR, 1.27; 95% CI, 1.04–1.56; *p* = 0.0218), 2‐tetradecenoyl carnitine (OR, 1.19; 95% CI, 1.01–1.41; *p* = 0.0394), X‐13431‐nonanoylcarnitine (OR, 1.24; 95% CI, 1.00–1.54; *p* = 0.0499), and piperine (OR, 0.83; 95% CI, 0.71–0.97; *p* = 0.0178) (Figure 3). However, after applying the Benjamini–Hochberg adjustment, none of these metabolites retained statistical significance (Table S3). Therefore, further investigation is warranted for these blood metabolites in subsequent analyses.

### 3.3 Reverse MR Analysis

The use of the IVW model showed no evidence of a causal effect of acne on the candidate metabolites (Table S8).

### 3.4 Multivariable MR Analysis

**FIGURE 4:**
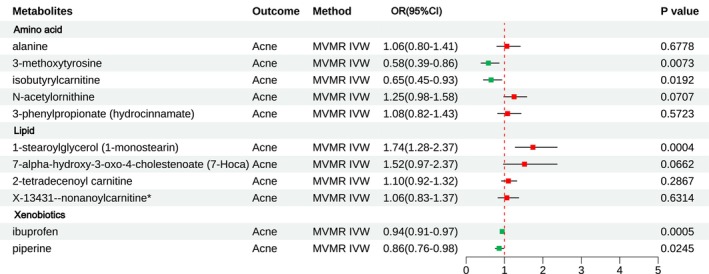
Forest plot presenting results from multivariable MR analysis. IVW, inverse‐variance weighted; OR, odds ratio.

To consider potential interactions among metabolites within the same category, we conducted a multivariable MR analysis (Table S10). Among the analyzed amino acids, 3‐methoxytyrosine (OR, 0.58; 95% CI, 0.39–0.86; *p* = 0.0073) and isobutyrylcarnitine (OR, 0.65; 95% CI, 0.45–0.93; *p* = 0.0192) showed significant inverse associations with acne, even after adjusting for potential confounding factors, including alanine, *N*‐acetylornithine, and 3‐phenylpropionate. Within the Lipid class, 1‐stearoylglycerol (OR, 1.74; 95% CI, 1.28–2.37; *p* = 0.0004) demonstrated a significant positive association with acne, even after adjusting for potential confounding factors, including 7‐Hoca, 2‐tetradecenoyl carnitine, and nonanoylcarnitine. In the Xenobiotics category, ibuprofen (OR, 0.94; 95% CI, 0.91–0.97; *p* = 0.0005) and piperine (OR, 0.86; 95% CI, 0.76–0.98; *p* = 0.0245) exhibited significant negative associations with acne (Figure 4).

### 3.5 Genetic Correlation Analysis

The LDSC analysis revealed a significant genetic correlation between nonanoylcarnitine and acne (rg = 2.0641, *p* = 0.0171), making it the most notable finding among all examined metabolites (Table S11).

### 3.6 Genetic Colocalization Analysis

**FIGURE 5:**
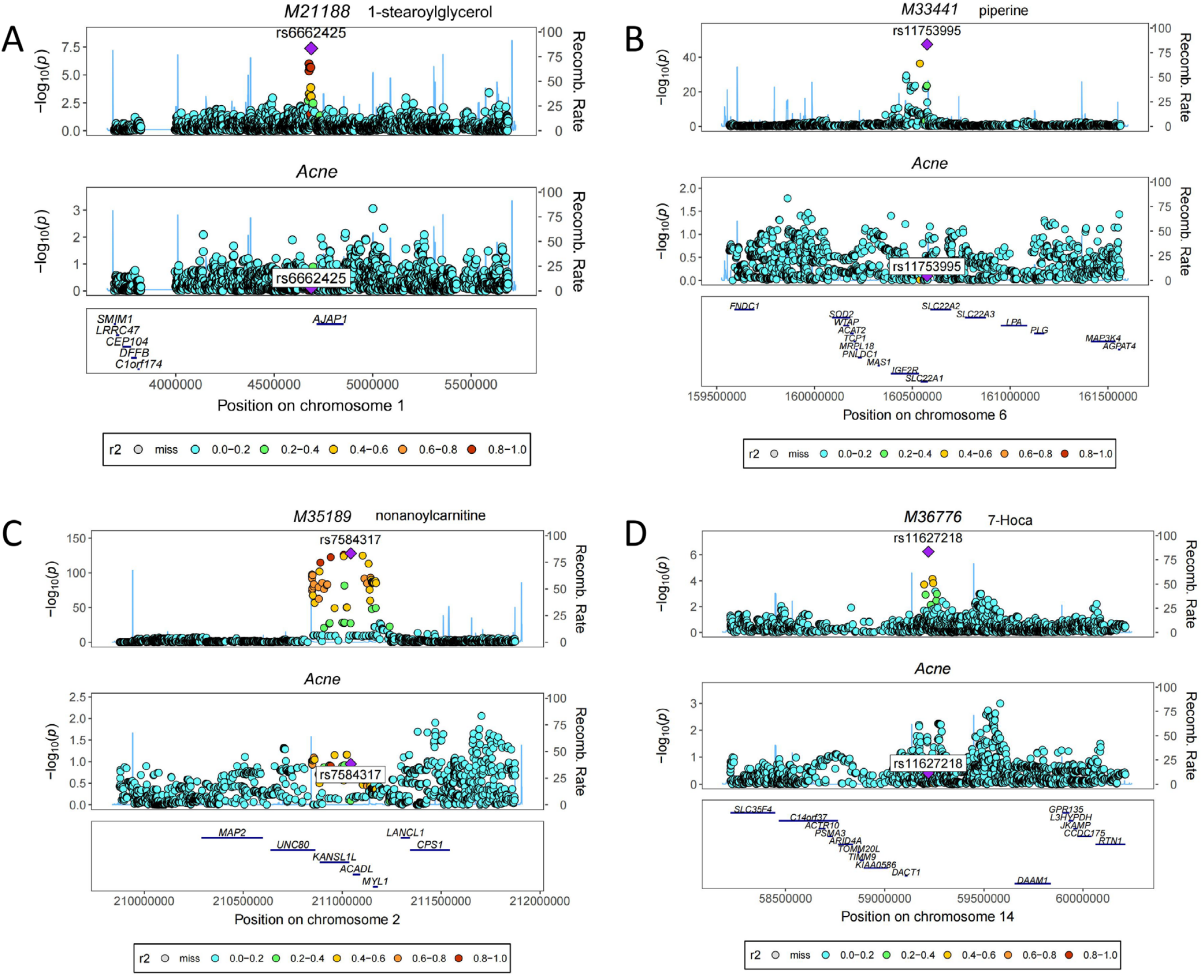
Colocalization analysis of candidate blood metabolites related to acne. Plots show genetic evidence of colocalization between acne and (A) 1‐stearoylglycerol at chr1:4688033; (B) isobutyrylcarnitine at chr6:160575366; (C) nonanoylcarnitine at chr2:211043102; (D) 7‐Hoca at chr14:59219801. Each point represents a single‐nucleotide polymorphism (SNP), with the chromosomal position on the *x*‐axis (within 500‐kb regions of the lead SNP) and − log10 (*p*‐value) on the *y*‐axis. Variants are color‐coded by their linkage disequilibrium with the lead SNP. Recombination rates are shown as blue lines, and gene locations are marked at the bottom.

The colocalization analysis revealed specific metabolites, including 1‐stearoylglycerol, isobutyrylcarnitine, nonanoylcarnitine, and 7‐Hoca, to be associated with acne, with genetic variations at shared loci playing a significant role in their respective relationships (Figure 5) (Table S11).

### 3.7 Metabolic Pathway Analysis

Our analysis of known metabolites identified two potential metabolic pathways involved in acne development (Table S13). Specifically, the arginine biosynthesis pathway and the selenocompound metabolism pathway may represent key biological mechanisms in acne pathogenesis.

## 4 Discussion

Acne is a complex skin condition associated with various metabolic processes. Its relationship with metabolism may involve hormonal and microbial factors. The conversion of testosterone to dihydrotestosterone has been proposed as a pathogenic mechanism in acne, with different conversion rates noted between acne‐affected and normal tissues [27]. Additionally, sebaceous glands in acne patients exhibit altered androgen metabolism compared to those without the condition [28]. Research has also explored the role of *Propionibacterium acnes* in acne comedones and its effects on neutrophil oxygen metabolism and phagocytosis, providing insights into systemic treatment options for acne [29].

Recent studies have highlighted the link between acne and blood metabolites. One study found that serum lipid levels and linoleic acid were significantly higher in acne patients, while levels of three essential and two nonessential amino acids were notably lower [30]. Nontargeted metabolomics has been used to explore the effects of isotretinoin on skin metabolism in acne‐affected rabbits, providing insights into relevant metabolic pathways [31]. However, it is important to note that research on blood metabolites and acne is still limited, with no studies examining their causal relationship. To our knowledge, this study is the first MR analysis investigating the causal link between blood metabolites and acne.

Our preliminary IVW analysis identified 12 eligible known metabolites that met strict screening criteria. These include 1‐stearoylglycerol, alanine, 3‐methoxytyrosine, isobutyrylcarnitine, ibuprofen, *N*‐acetylornithine, bilirubin, 7‐Hoca, 3‐phenylpropionate, 2‐tetradecenoyl carnitine, nonanoylcarnitine, and piperine. The selection was refined through sensitivity analyses, multivariable MR analysis, LDSC analysis, and colocalization analysis, identifying key metabolites significantly associated with acne.

Our study reveals a significant genetic correlation between nonanoylcarnitine and acne through LDSC analysis, providing valuable insights into the complex genetic factors involved in acne. Nonanoylcarnitine, a medium‐chain acylcarnitine, has been noted in metabolomic research, and its genetic influences have been explored across different platforms [32]. Genetic deletion of G protein–coupled receptor 27 (Gpr27) has been shown to elevate nonanoylcarnitine levels, suggesting its role in insulin sensitivity and glucose homeostasis in zebrafish [33]. The association between nonanoylcarnitine and acne may help elucidate the metabolic pathways involved in acne pathogenesis.

In this study, the multivariable MR analysis revealed nuanced insights into the relationships between selected metabolites and acne. Among the amino acids, 3‐methoxytyrosine and isobutyrylcarnitine exhibited significant inverse associations with acne. Within the lipid class, 1‐stearoylglycerol demonstrated a positive association with acne. In the Xenobiotics category, ibuprofen and piperine were found to be inversely associated with acne. Colocalization analysis confirmed shared genetic variants between four metabolites (1‐stearoylglycerol, isobutyrylcarnitine, nonanoylcarnitine, and 7‐Hoca) and acne. The colocalization analysis results and multivariable MR analysis showed an overlap in identified metabolites, thereby highlighting the significance of these metabolites in the pathogenesis of acne. 1‐Stearoylglycerol is a monoglyceride that has been associated with hepatic insulin resistance and has shown an inverse correlation with the risk of prostate cancer [34, 35]. Isobutyrylcarnitine is an acylcarnitine associated with specific metabolic disorders, including SCAD and isobutyryl‐CoA dehydrogenase deficiency [36]. However, no previous studies have investigated the relationship between these two metabolites and acne. The observed positive correlations between 1‐stearoylglycerol and acne, as well as the inverse associations between isobutyrylcarnitine and acne, underline the importance of further exploring these relationships in future studies.

This MR analysis investigates the relationship between 486 blood metabolites and acne, presenting several strengths. It is the first systematic examination of causal links, utilizing robust methods such as IVW analysis, sensitivity analysis, multivariable MR analysis, LDSC analysis, and colocalization analysis. These rigorous techniques addressed issues like reverse causality and confounding. Notably, LDSC analysis uncovered a genetic correlation between nonanoylcarnitine and acne, enhancing our understanding of acne's complex genetic landscape.

Previous research has highlighted associations between acne and blood metabolites, such as elevated serum lipid levels and linoleic acid, and reduced levels of both essential and nonessential amino acids in acne patients [30]. These studies have provided insights into the metabolic mechanisms underlying acne. Our study extends this knowledge by identifying specific metabolites associated with acne through comprehensive analyses. We found that nonanoylcarnitine and 1‐stearoylglycerol are positively associated with acne, while isobutyrylcarnitine shows a significant inverse association. These findings not only confirm some previously identified associations but also uncover new relationships, advancing our understanding of the metabolic regulation mechanisms involved in acne.

However, there are limitations. The original GWAS data were sourced from individuals of European ancestry, potentially limiting applicability to other ethnic groups. The study focused on linear relationships, leaving nonlinear effects of metabolites to be explored further. New associations, including 1‐stearoylglycerol and isobutyrylcarnitine, require additional investigation and validation. Future research should include laboratory studies, clinical cohort analyses, and examinations in diverse populations to confirm and expand upon these findings.

## 5 Conclusion

In summary, our study conducted a comprehensive metabolome‐wide MR analysis, identifying 12 metabolites significantly associated with acne. The IVW analysis, complemented by sensitivity, multivariable MR, LDSC, and colocalization analyses, provided strong evidence of potential causal relationships between these metabolites and acne. Reverse MR analysis showed no causal effect of acne on these metabolites. We identified independent causal effects of specific metabolites, such as 1‐stearoylglycerol, isobutyrylcarnitine, and nonanoylcarnitine, on acne. Colocalization analysis confirmed shared genetic variants between four metabolites and acne, emphasizing their role in acne pathogenesis. Metabolic pathway analysis highlighted the arginine biosynthesis and selenocompound metabolism pathways as key mechanisms in acne development.

These findings enhance our understanding of the complex interactions between metabolism and acne, suggesting potential biomarkers for disease risk and progression. Our research not only refines diagnostic precision and informs personalized treatment strategies but also deepens the understanding of acne's molecular mechanisms. Integrating genetic and metabolomic data has illuminated crucial biological pathways, offering valuable insights for future research and therapeutic development.

## Author Contributions

M.L. and Z.Z. were responsible for the study conception and design. M.L., L.L.F., D.D.Z., and Y.W. performed data analysis and drafted the manuscript. X.H.H. and M.Z. managed data acquisition and study implementation. All authors reviewed and approved the final manuscript.

## Ethics Statement

All data utilized in this study were sourced from publicly accessible databases; obtaining additional ethical approval was not required.

## Consent

This study did not require ethics approval or informed consent as it utilized publicly available summary‐level data from genome‐wide association studies (GWAS). All original studies from which these data were derived had already obtained the necessary ethical approvals and informed consents.

## Conflicts of Interest

The authors declare no conflicts of interest.

## Supporting information


Figure S1.
Figure S2.

Table S1.
Table S2.
Table S3.
Table S4.
Table S5.
Table S6.
Table S7.
Table S8.
Table S9.
Table S10.
Table S11.
Table S12.
Table S13.

## Data Availability

This study utilized publicly available datasets for analysis. The mGWAS dataset can be obtained through the Metabolomics GWAS Server (http://metabolomics.helmholtz‐muenchen.de/gwas/). Specifically, the GWAS datasets for acne can be accessed from the GWAS Catalog (https://www.ebi.ac.uk/gwas/studies/GCST90245818).
