# Supplementary material for: Unraveling the Causal Relationship Between Blood Metabolites and Acne: A Metabolomic Mendelian Randomization Study
Source: J Cosmet Dermatol. 2024 Dec 31;24(1):e16763. doi: 10.1111/jocd.16763 (PMC11686570; doi:10.1111/jocd.16763)

**Supplementary Figure 1 Summary of scatter plots illustrating potential positive associations between blood metabolites and the risk of acne**


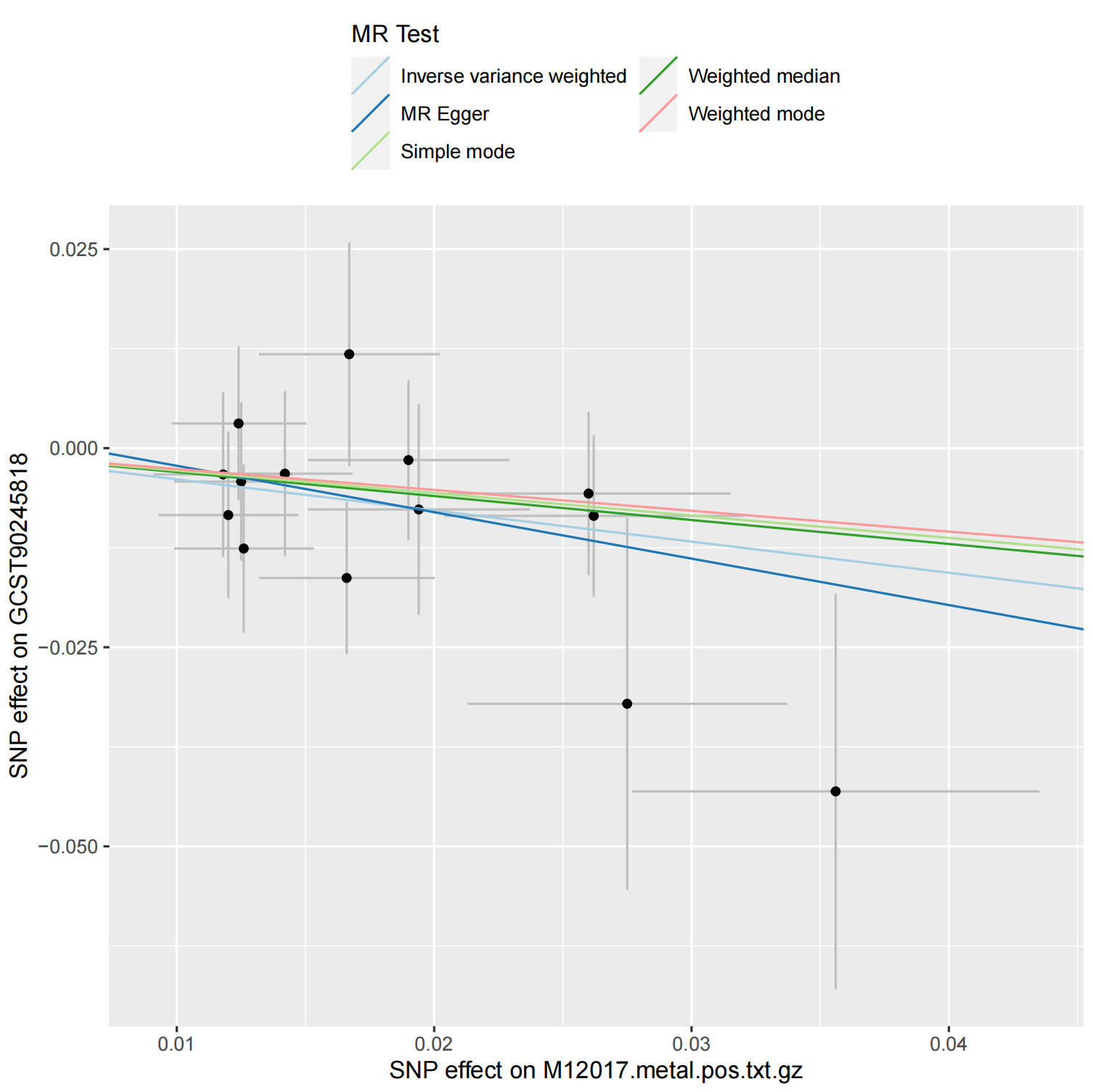

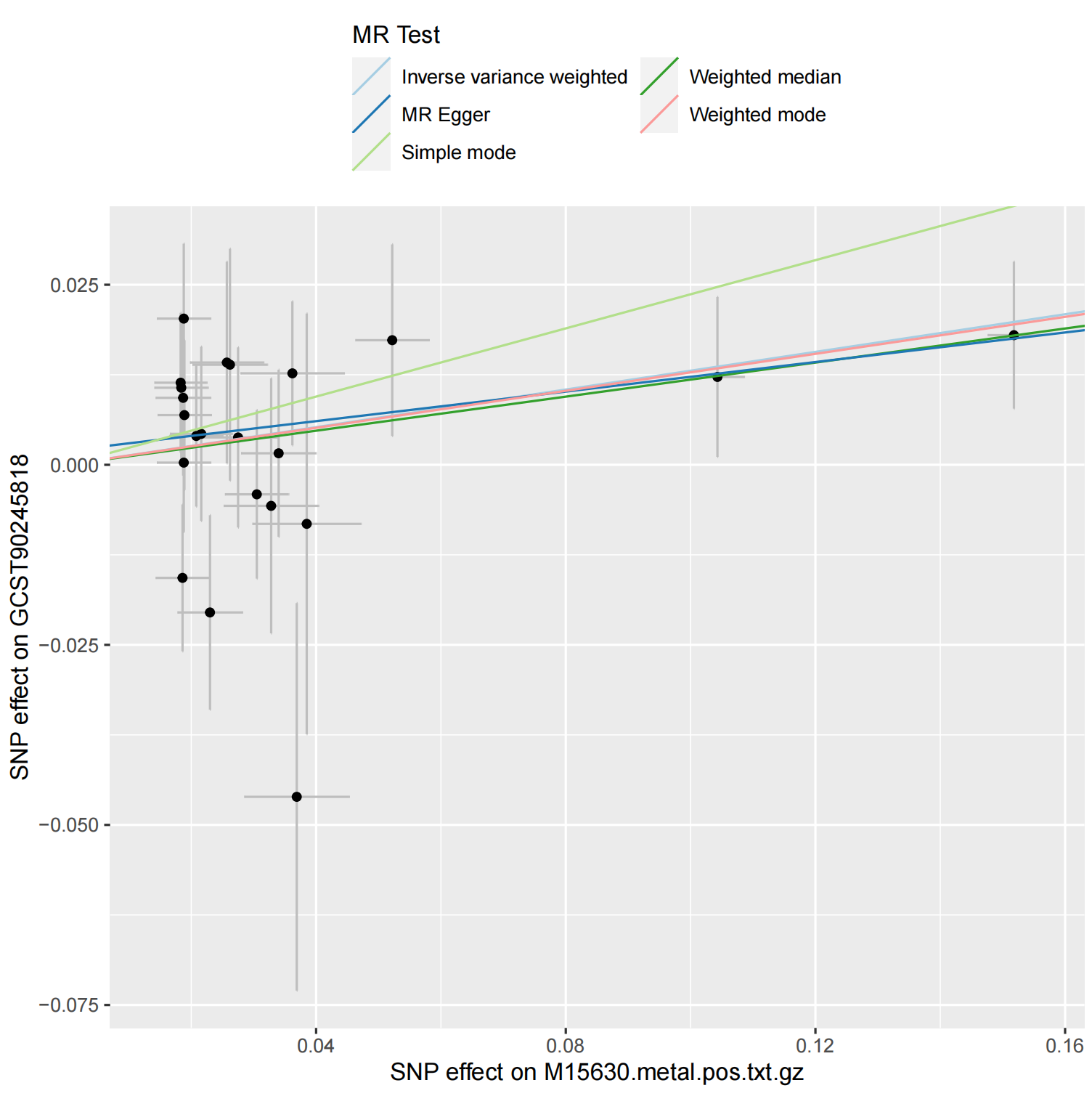


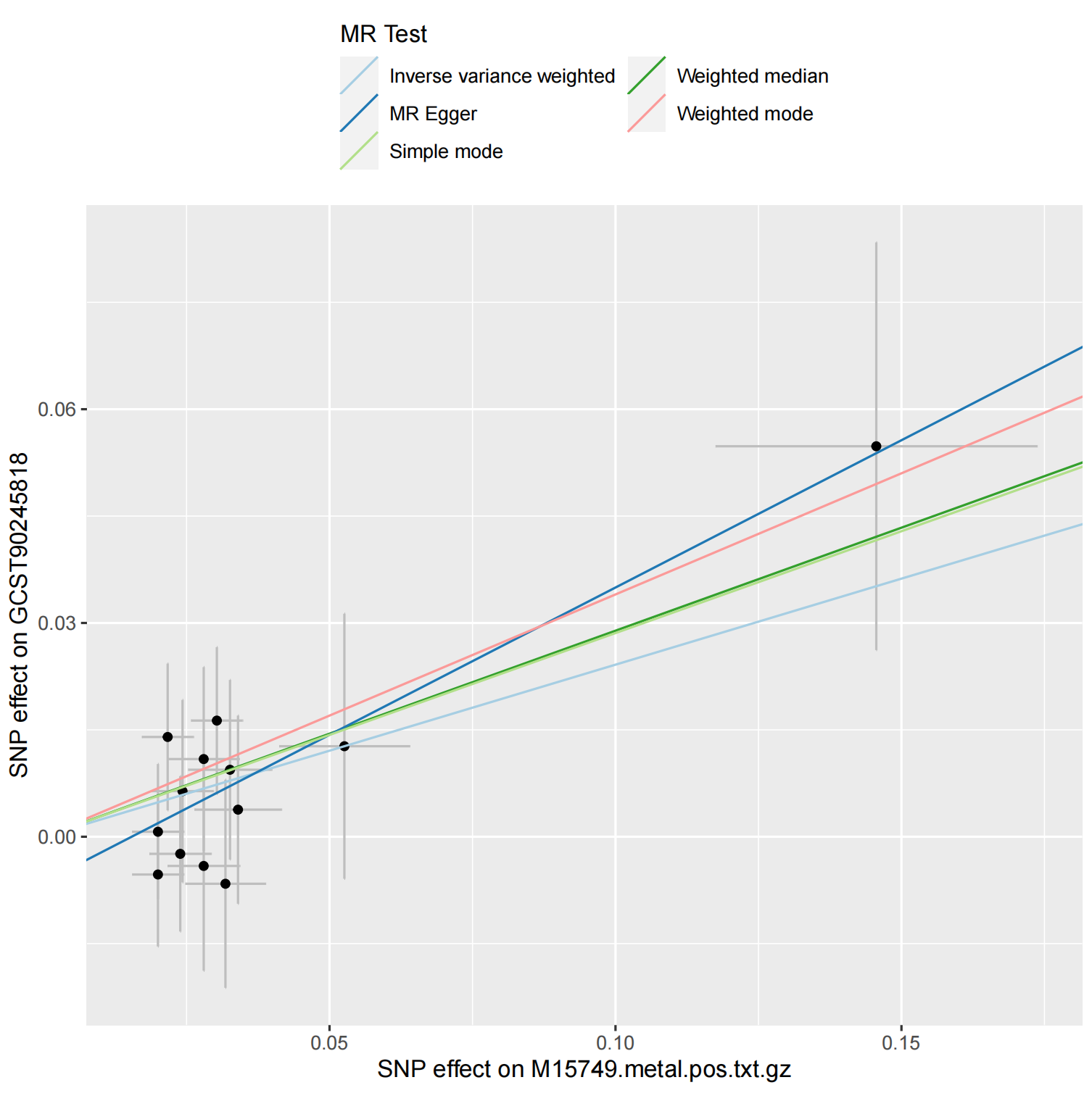

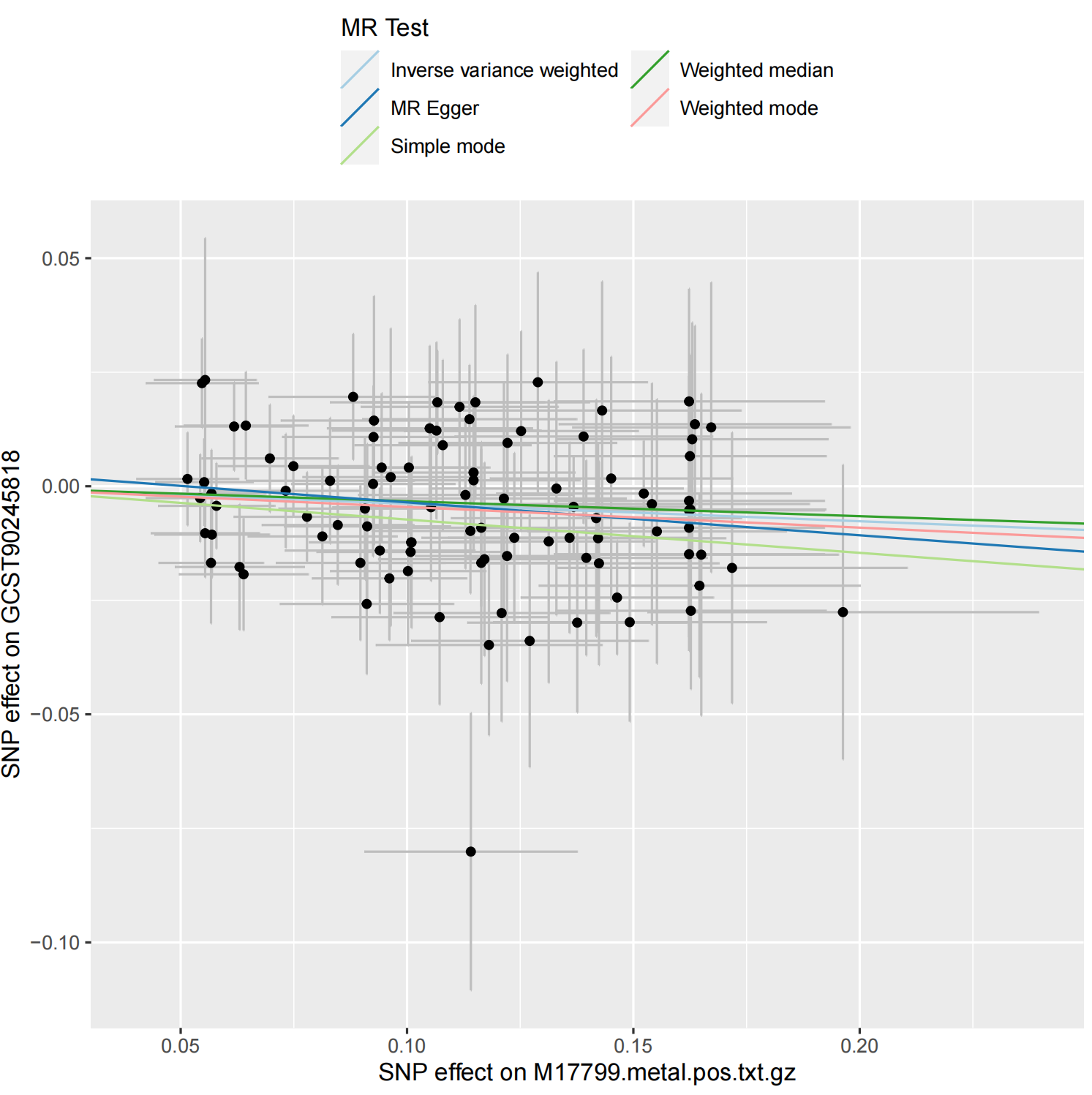


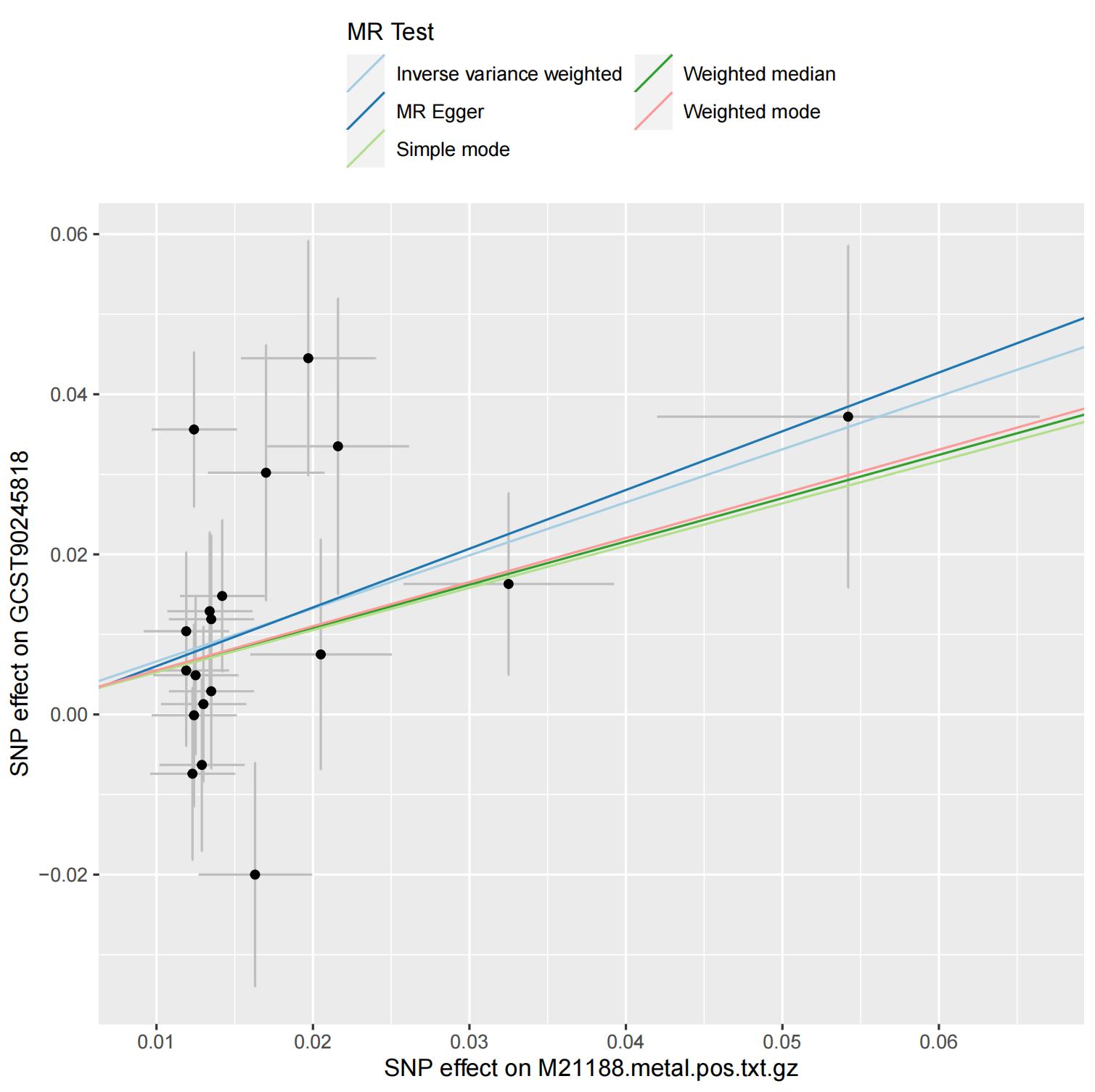

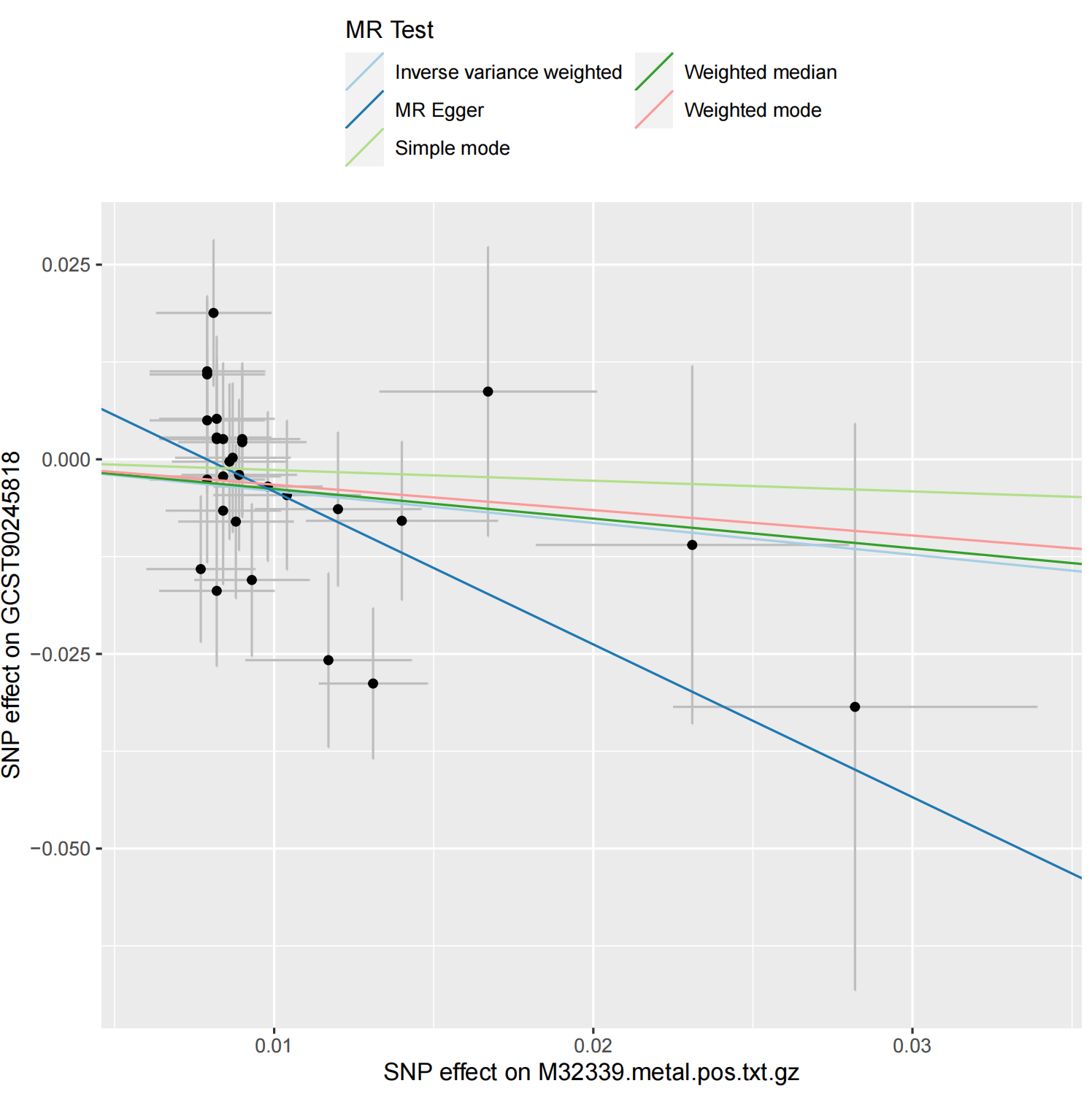


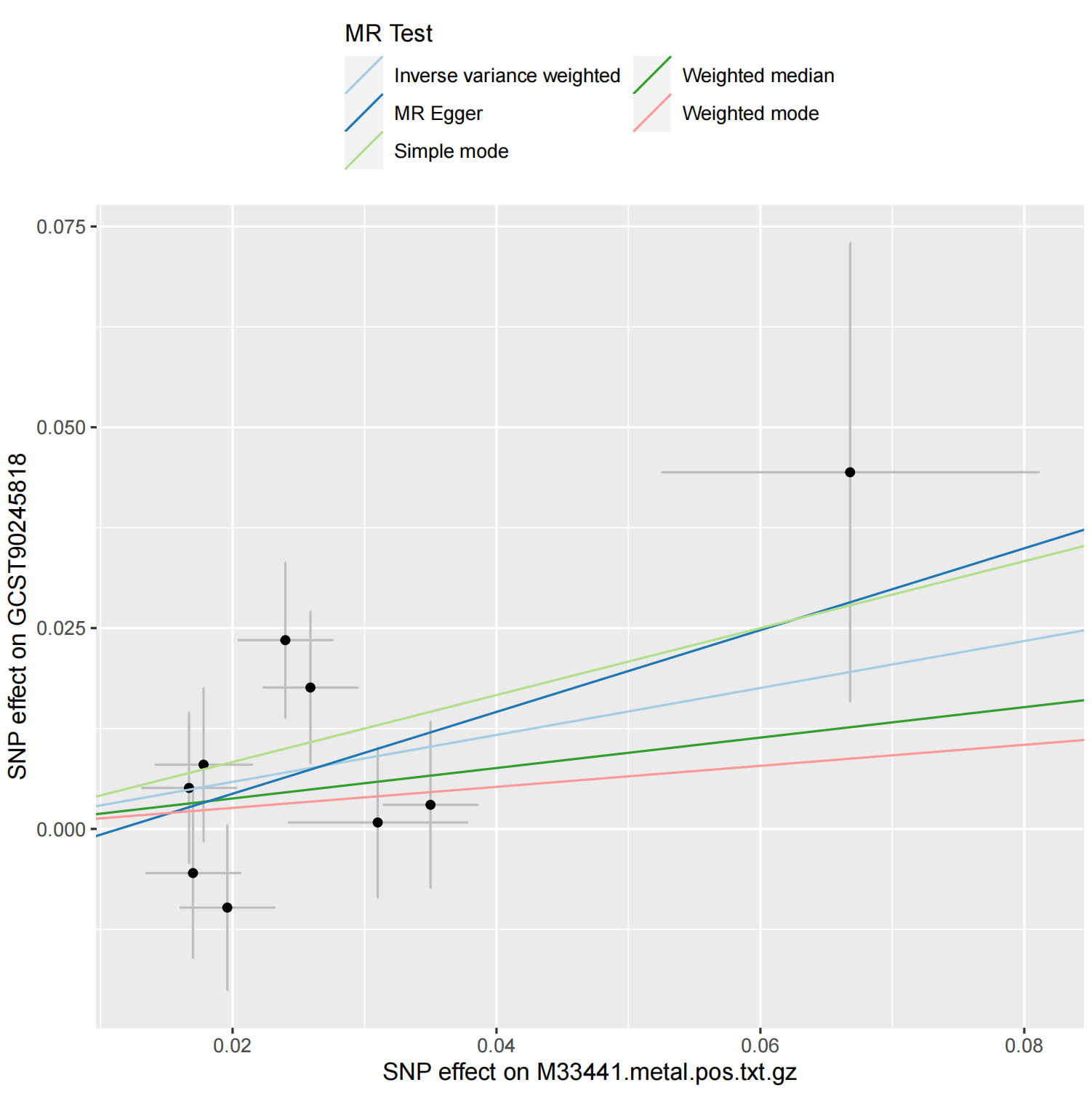

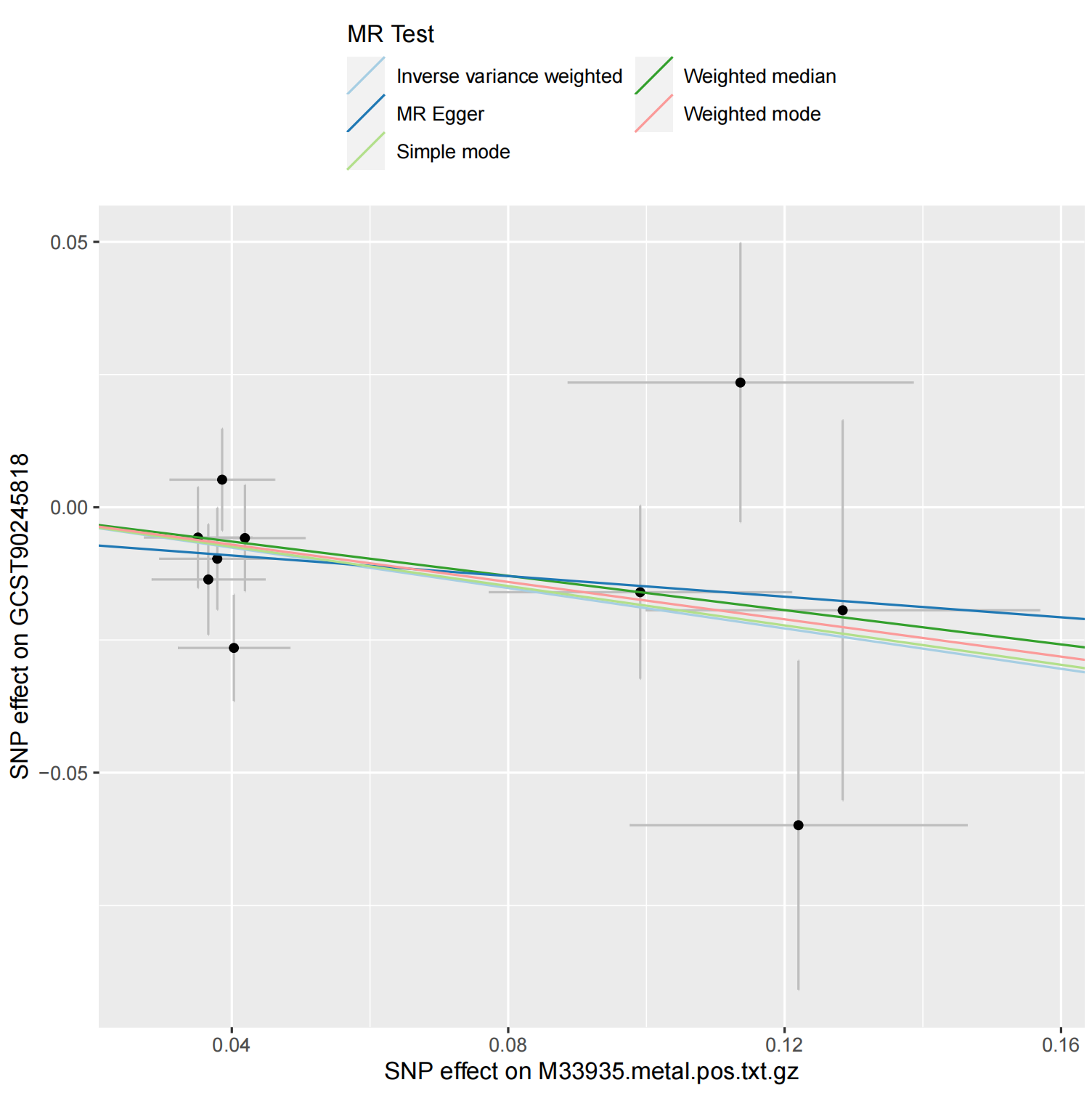


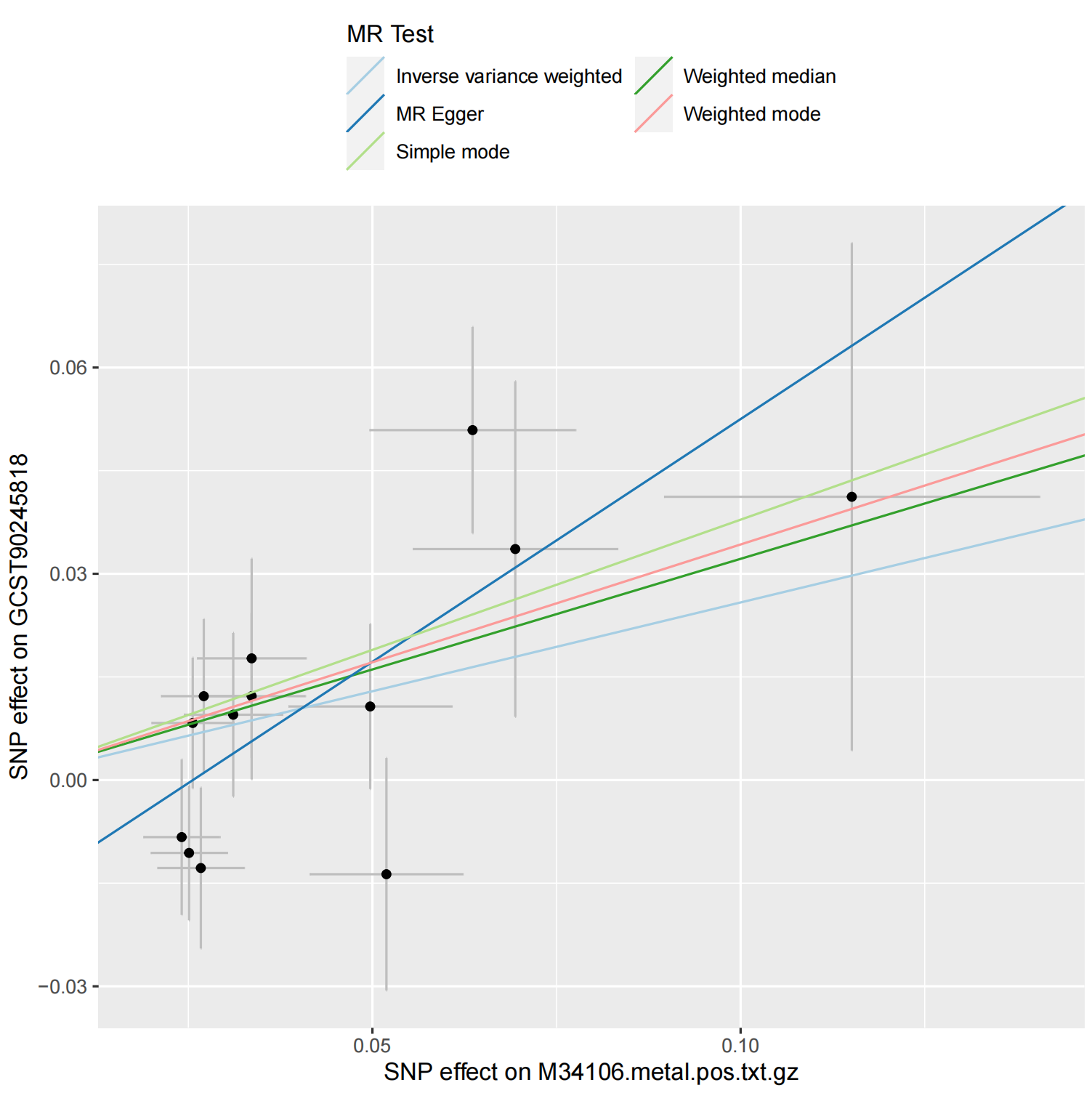

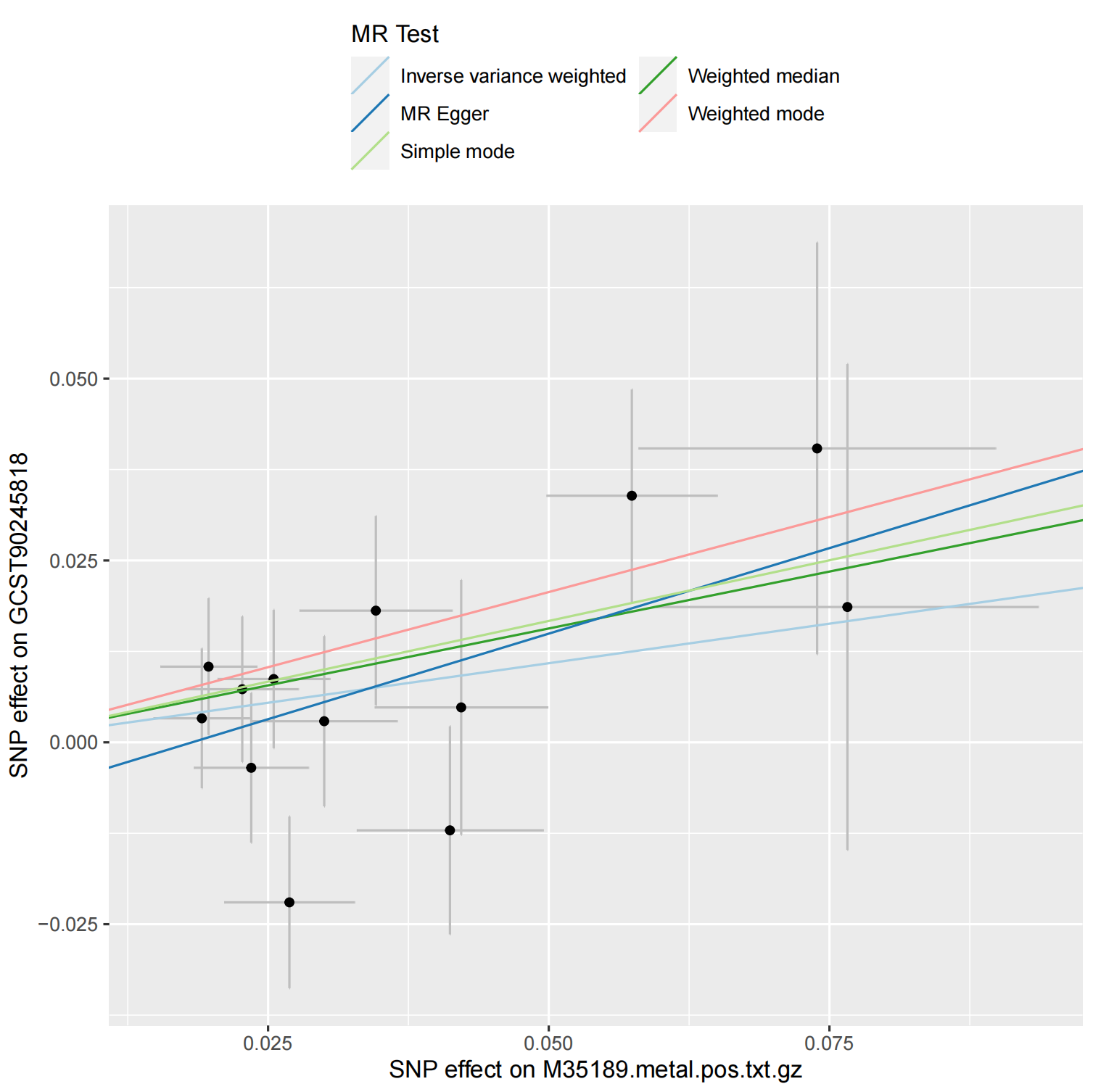


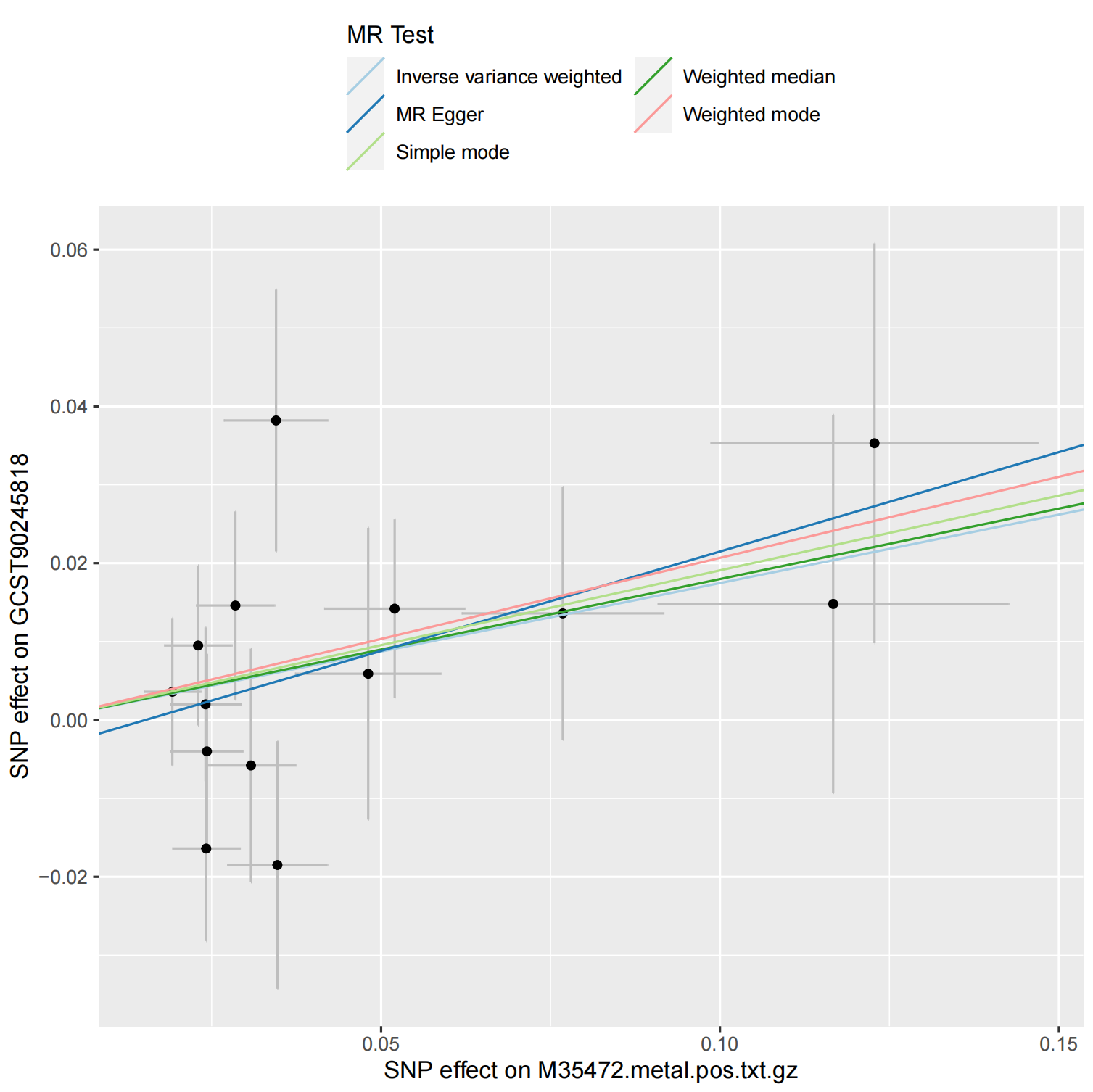

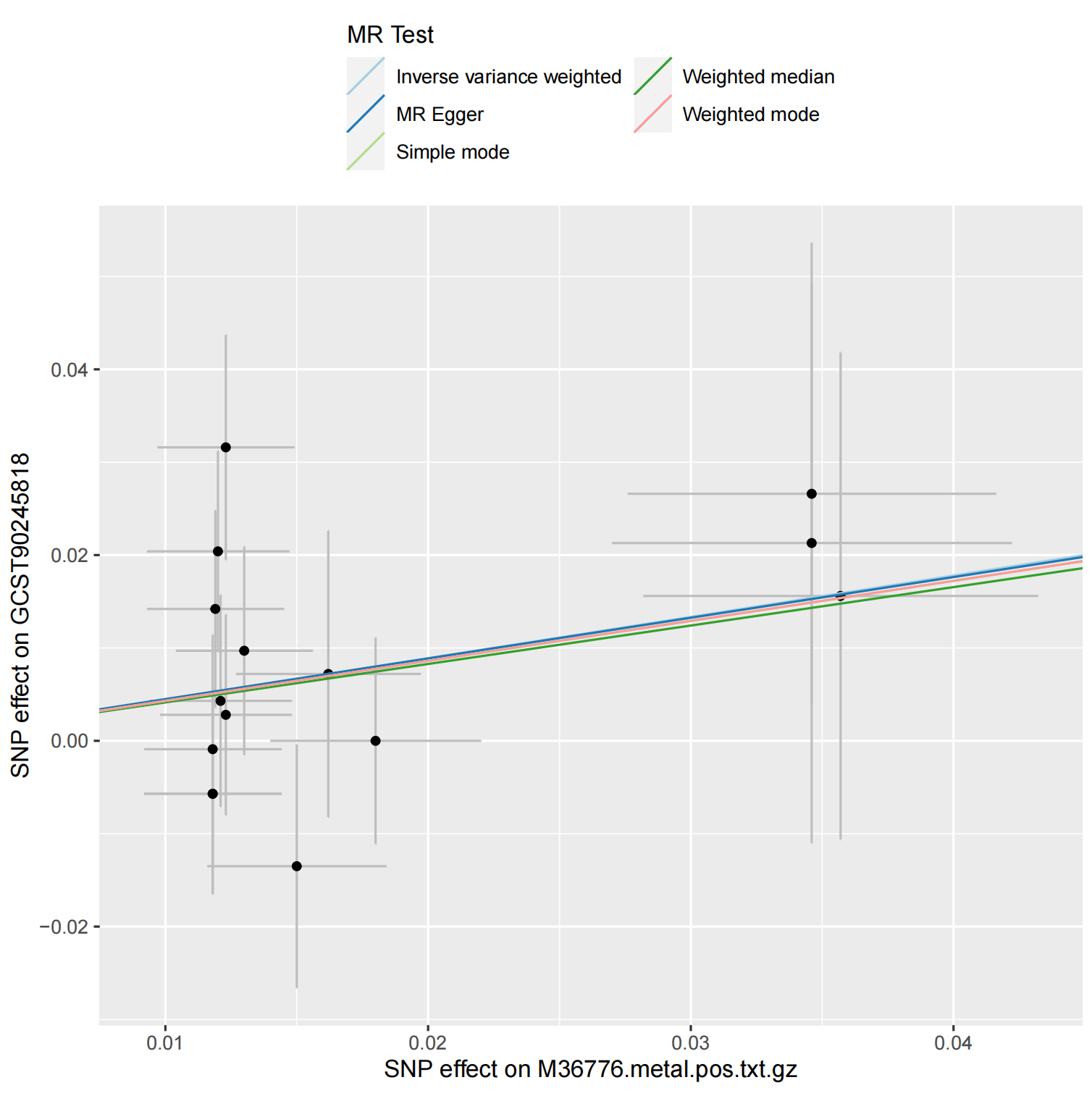


**Supplementary Figure 2 Forest plots for the Mendelian randomization (MR) leave-one-out analysis of the significant inverse variance weighted (IVW) estimates.**


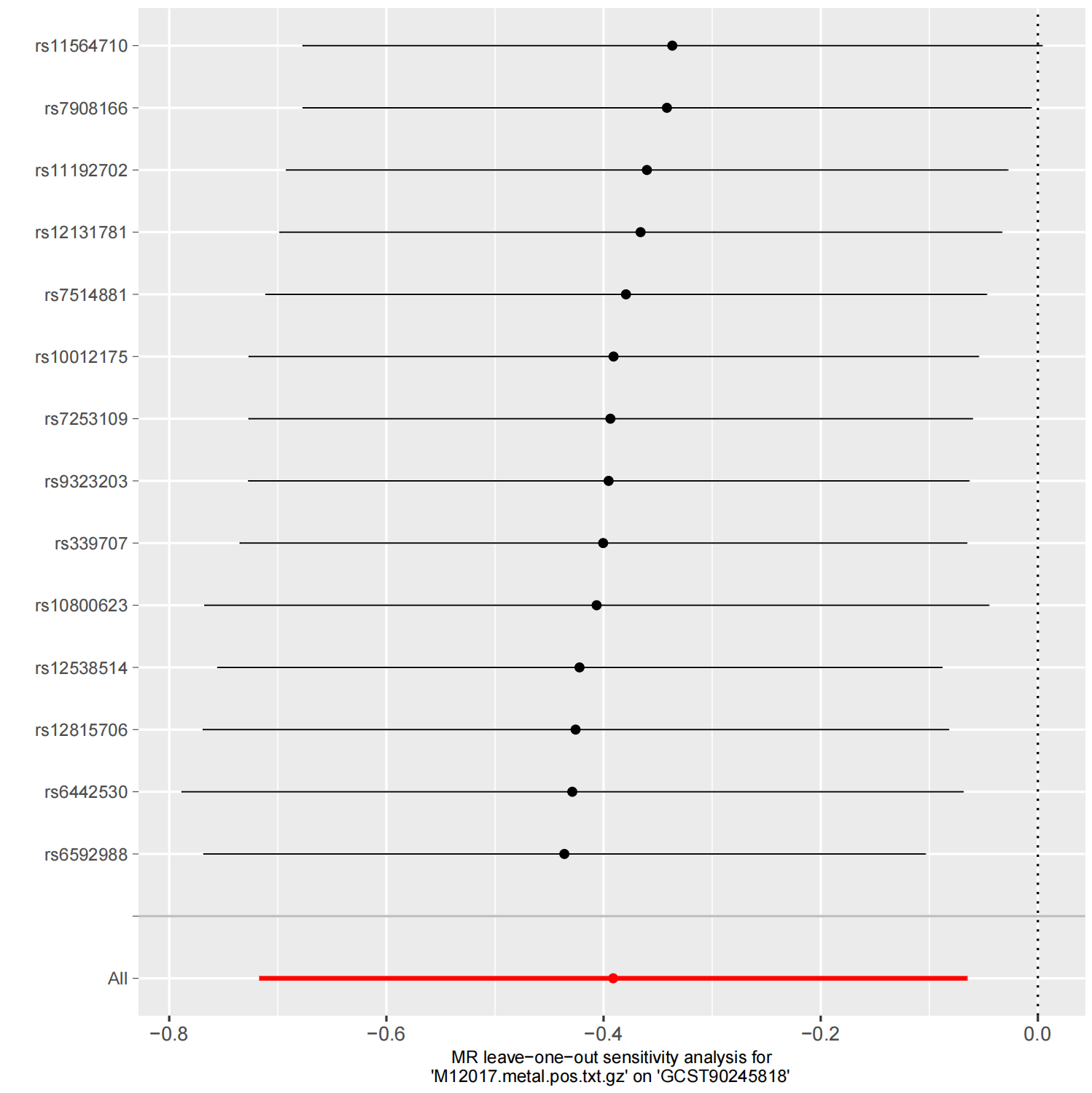

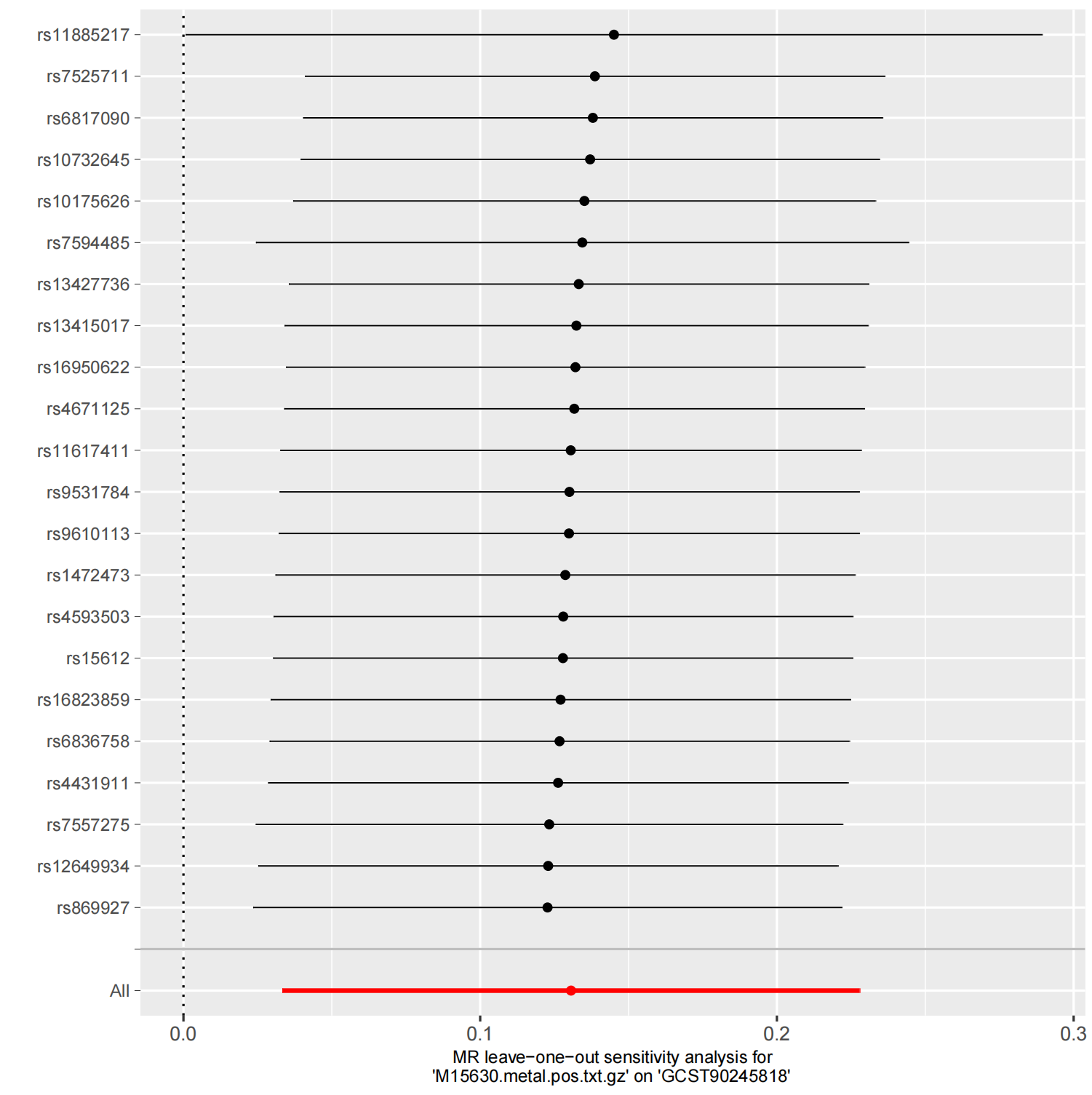


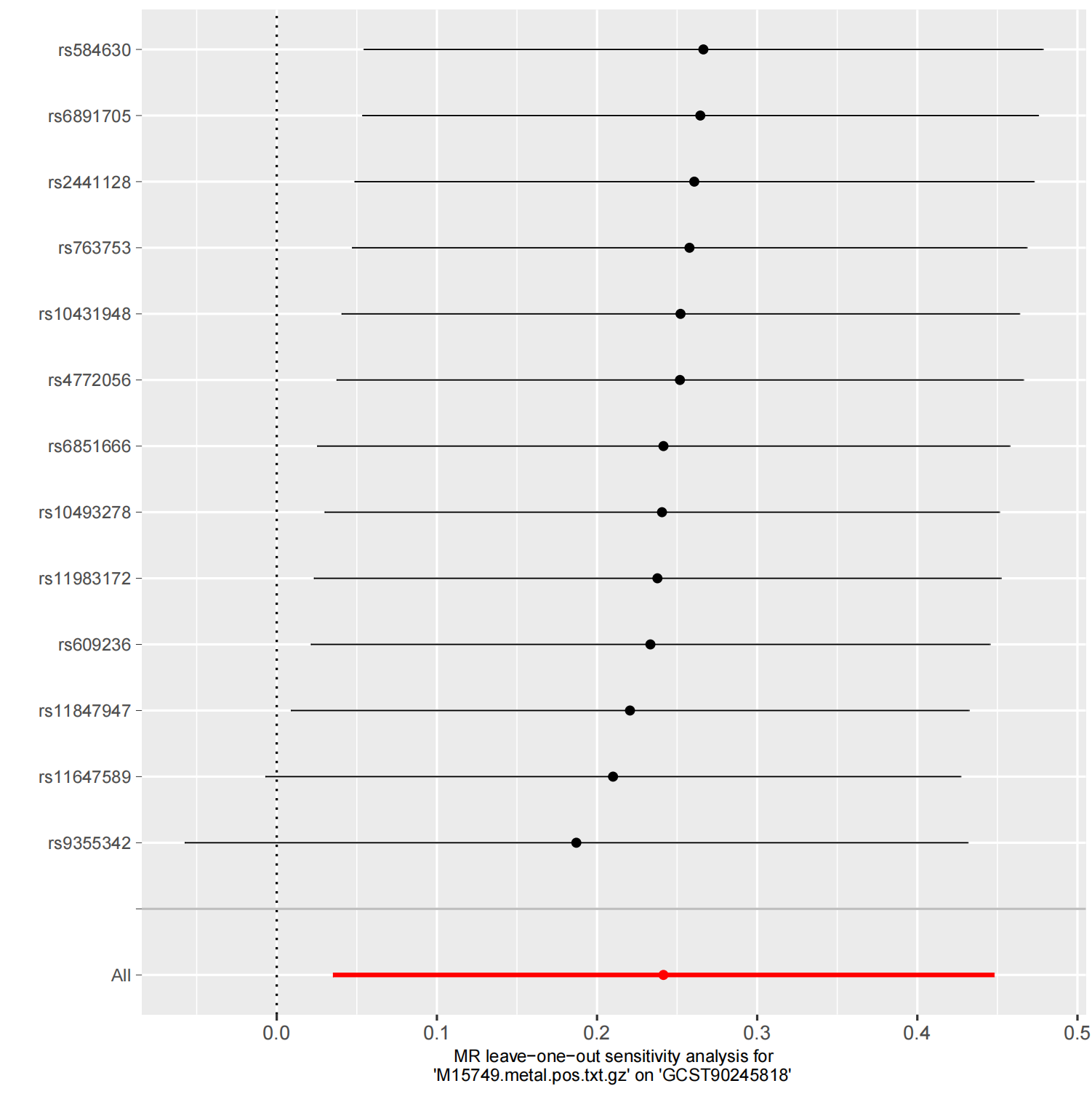

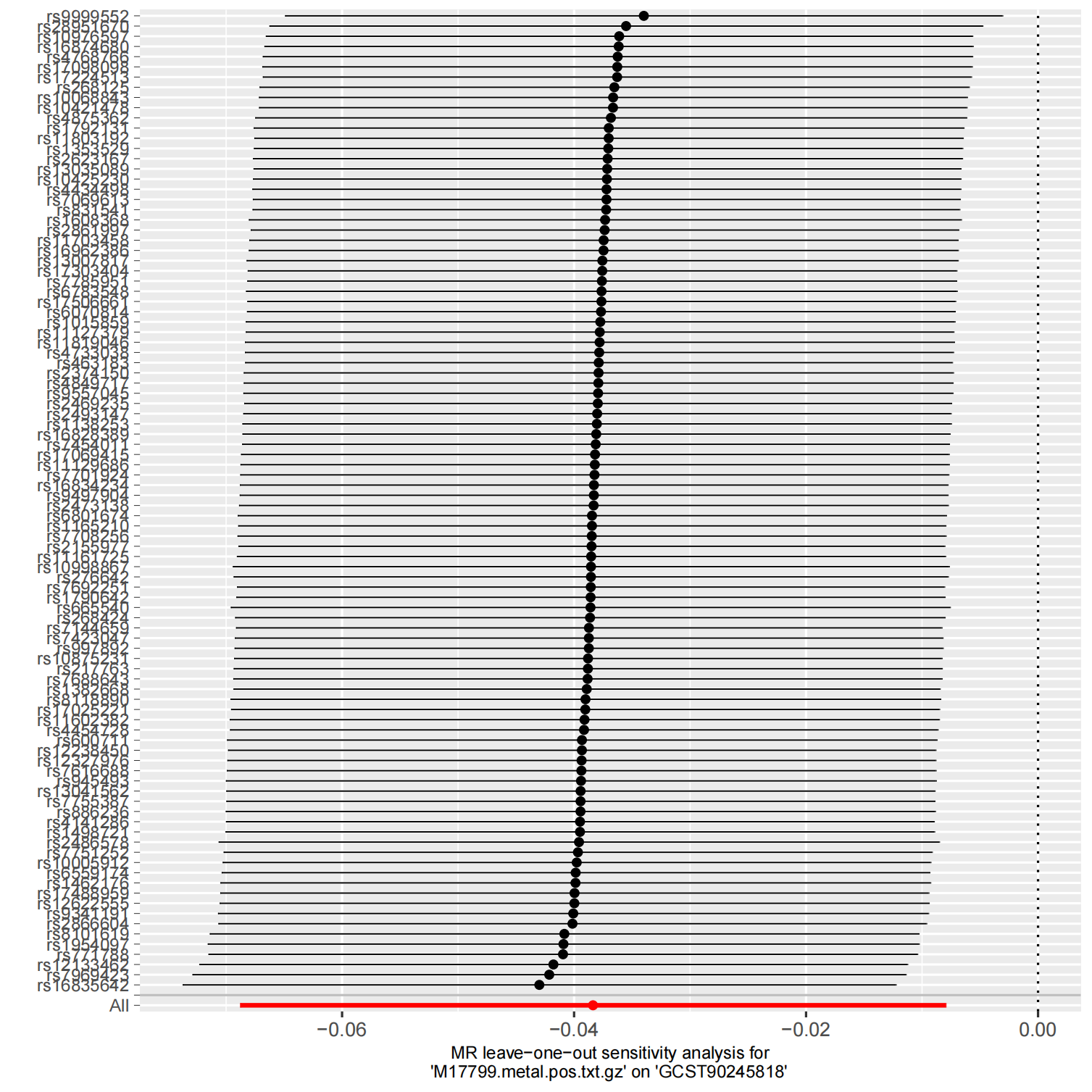


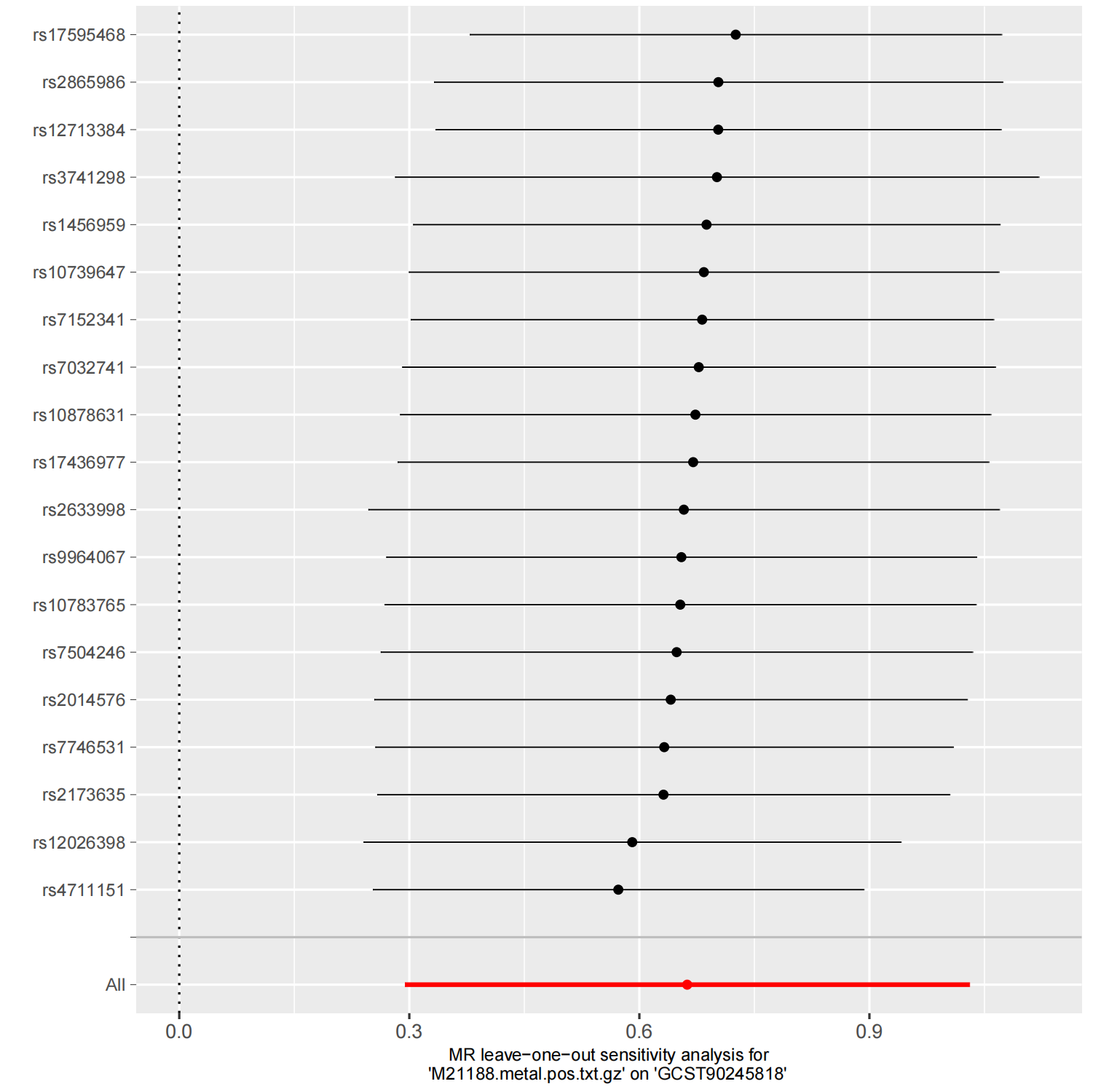

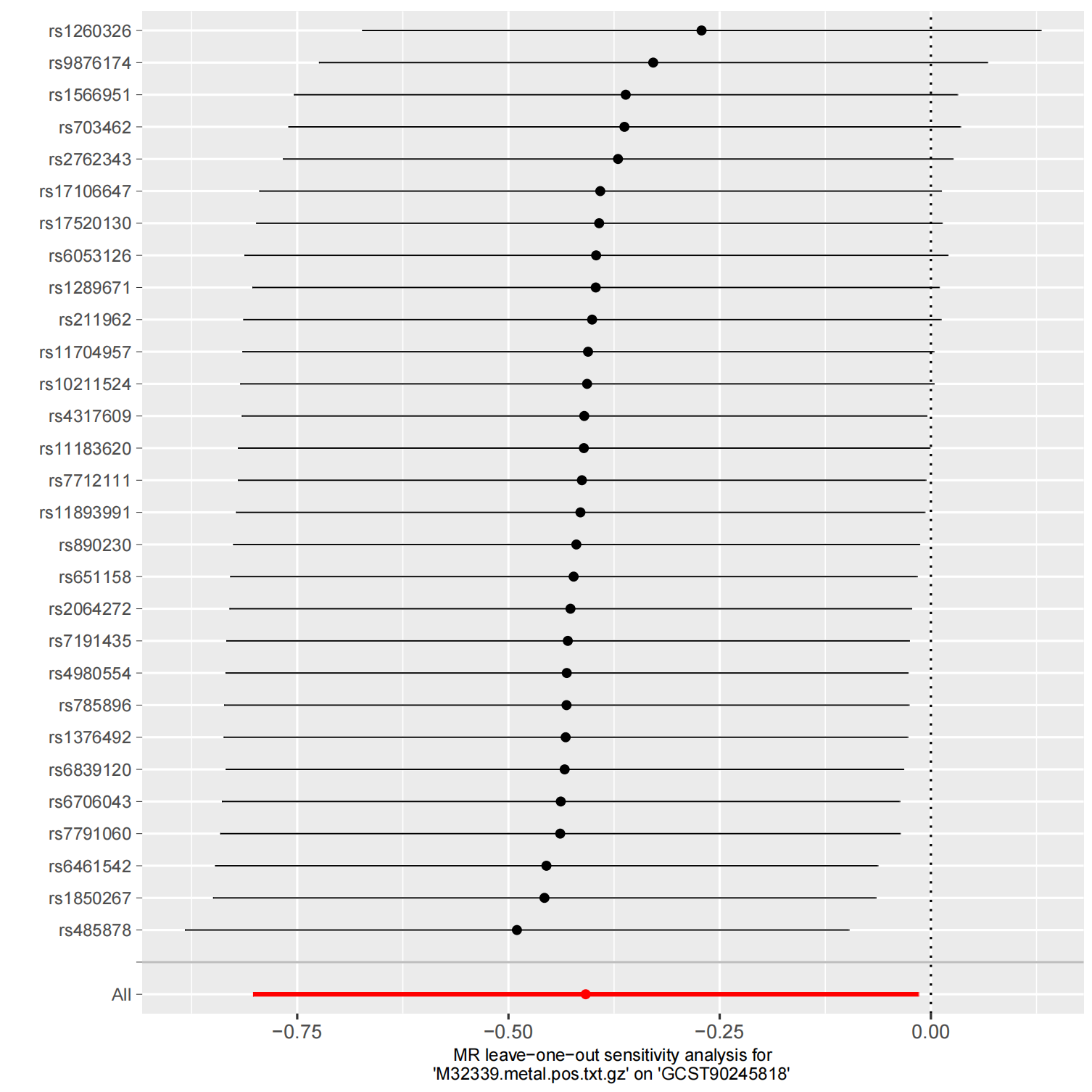


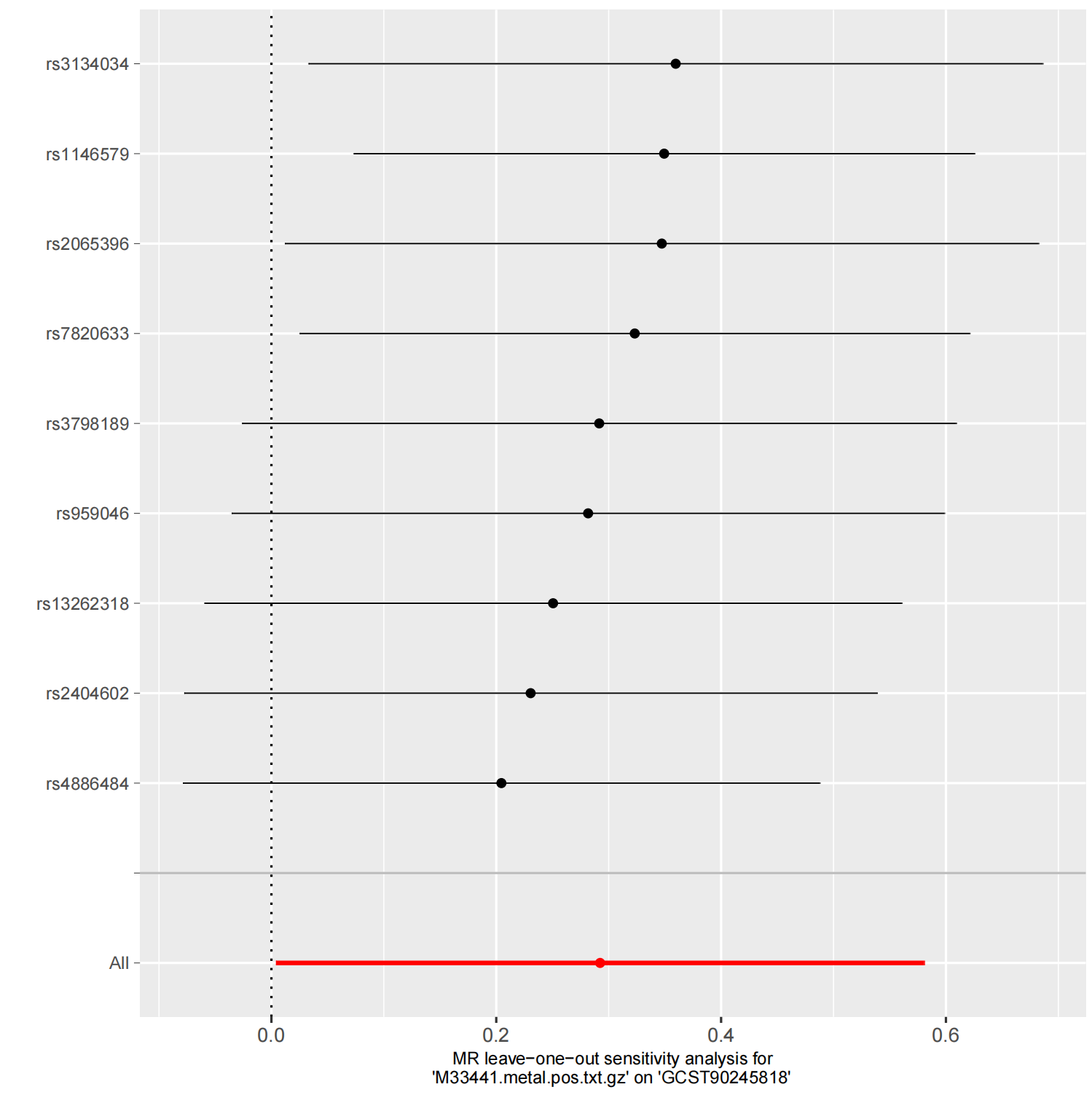

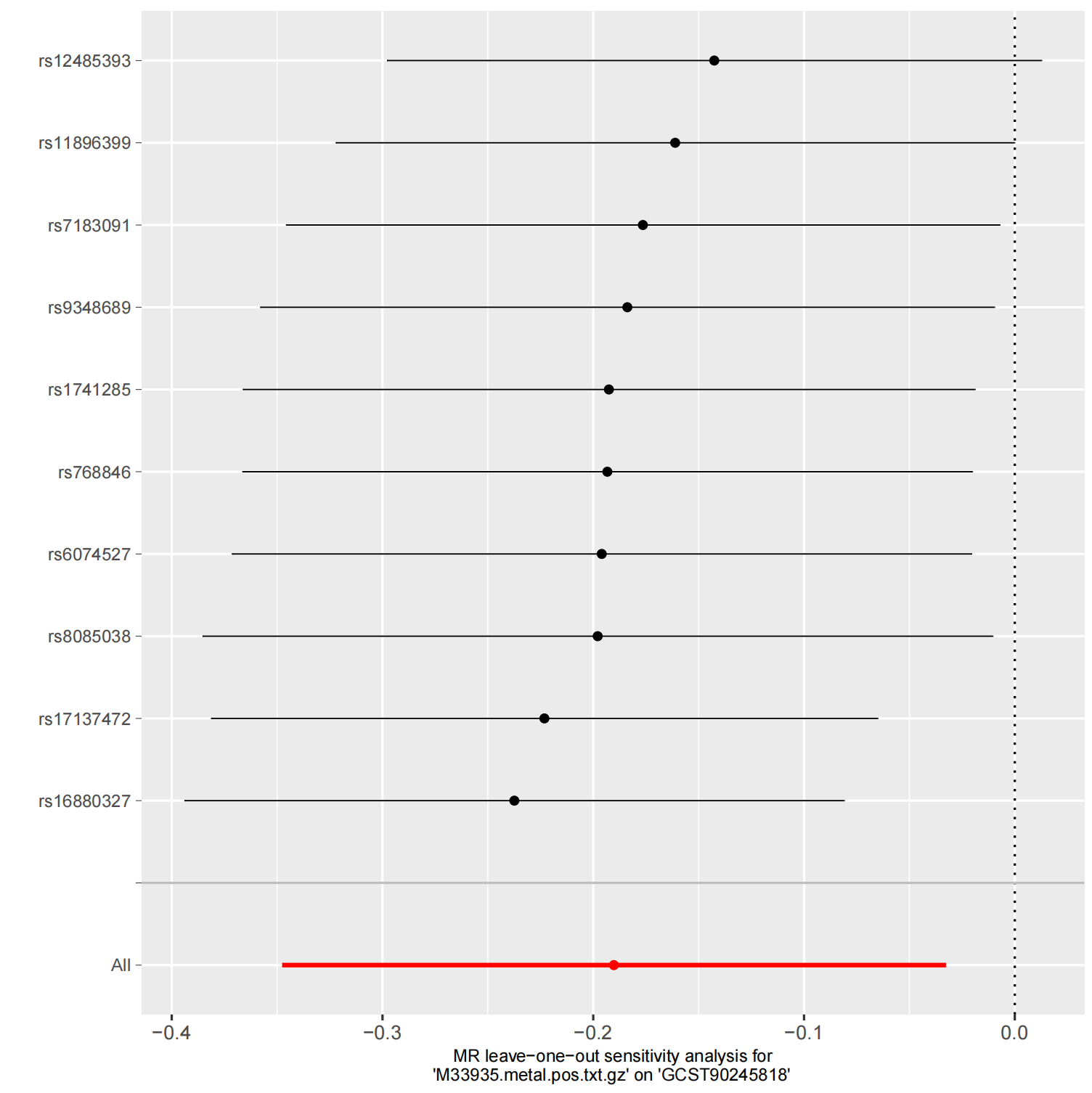

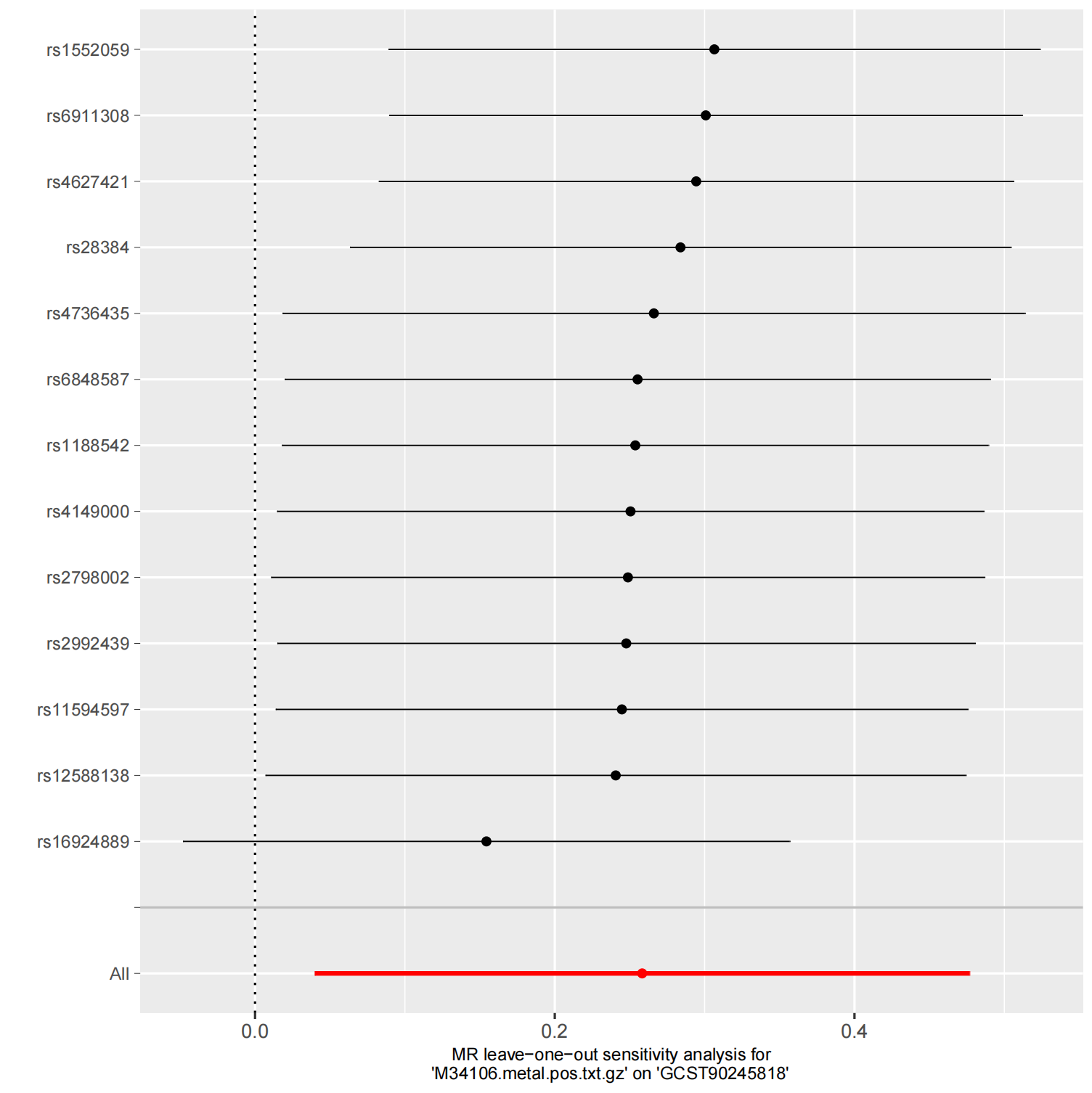

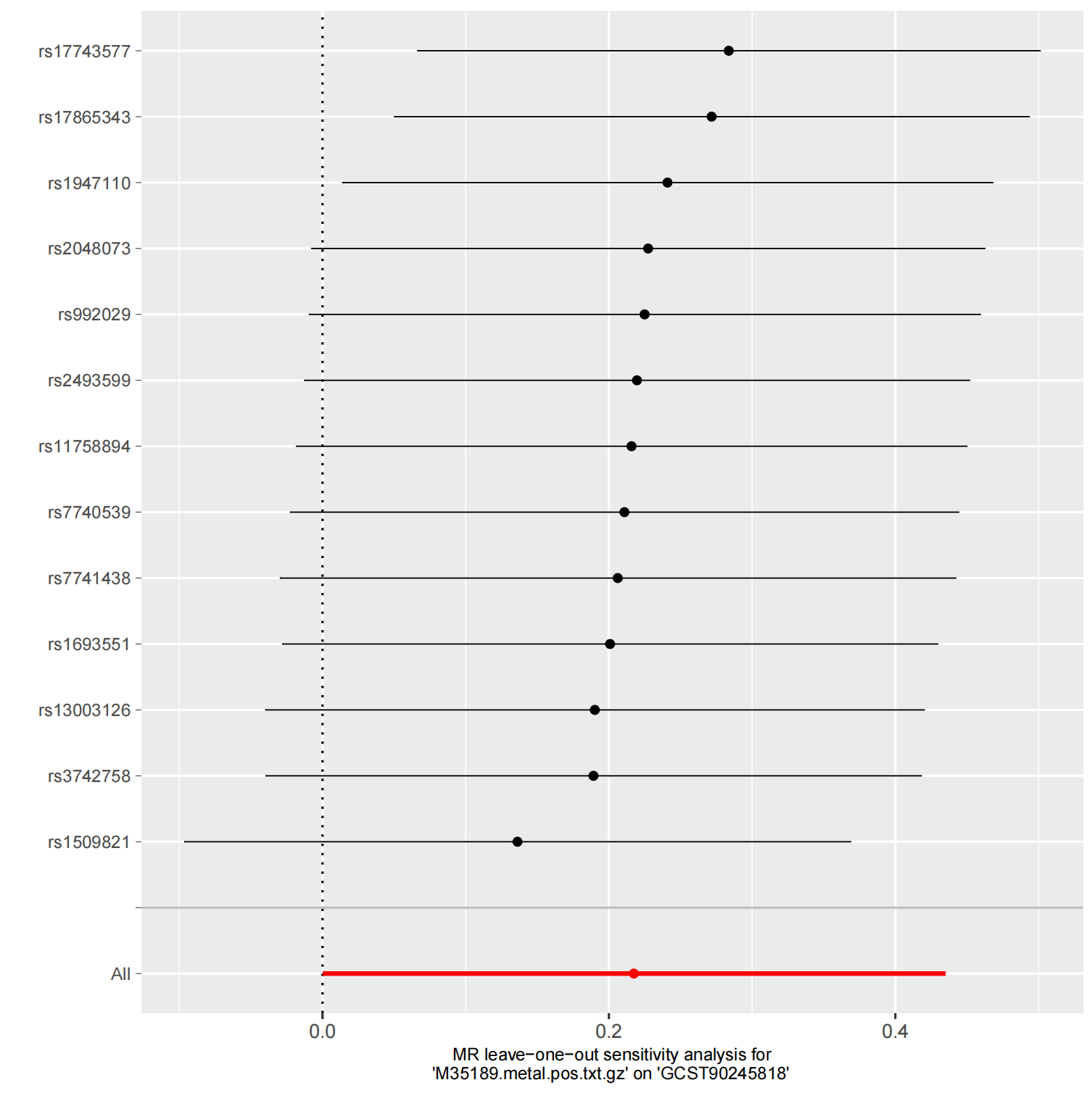


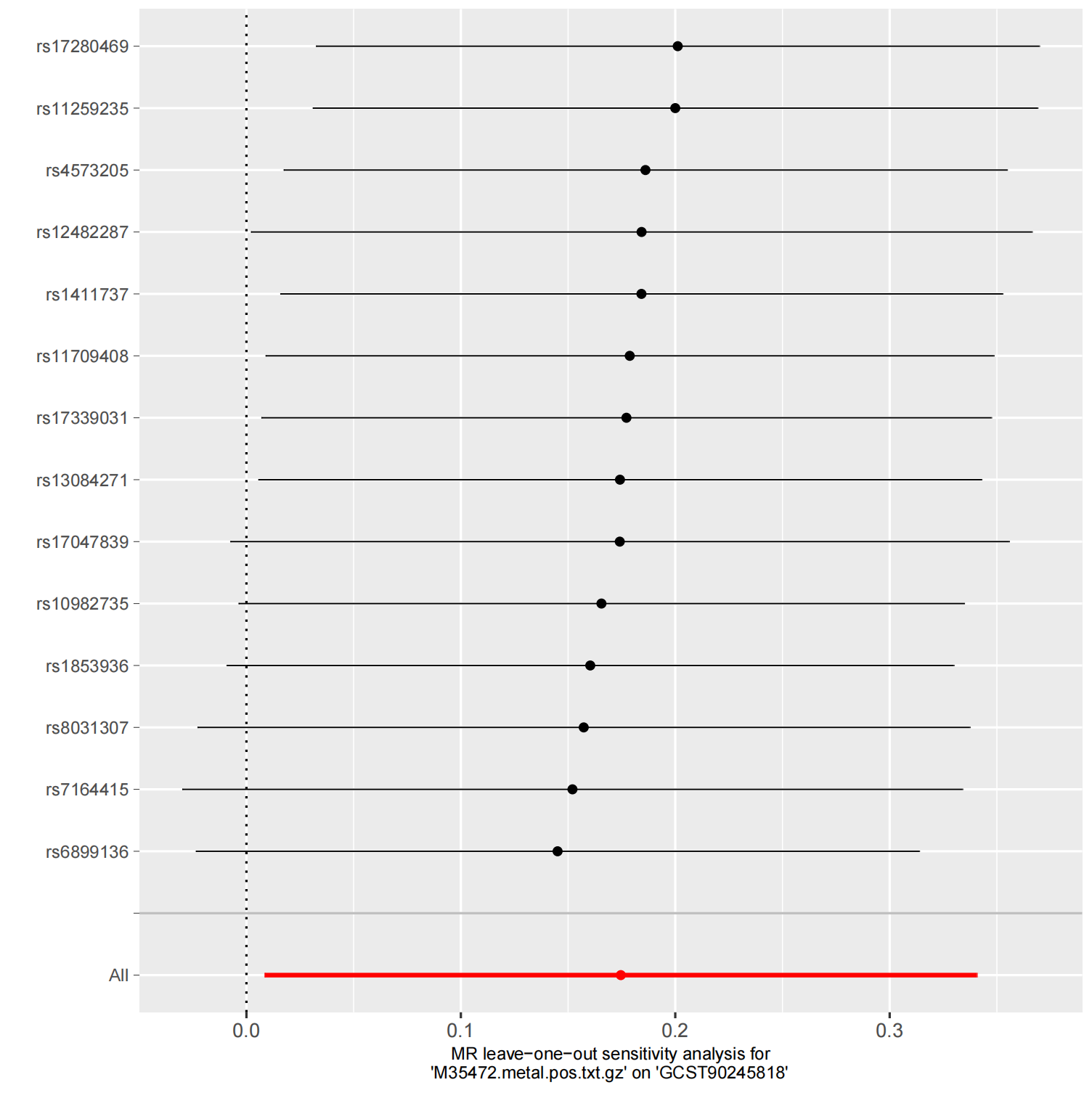

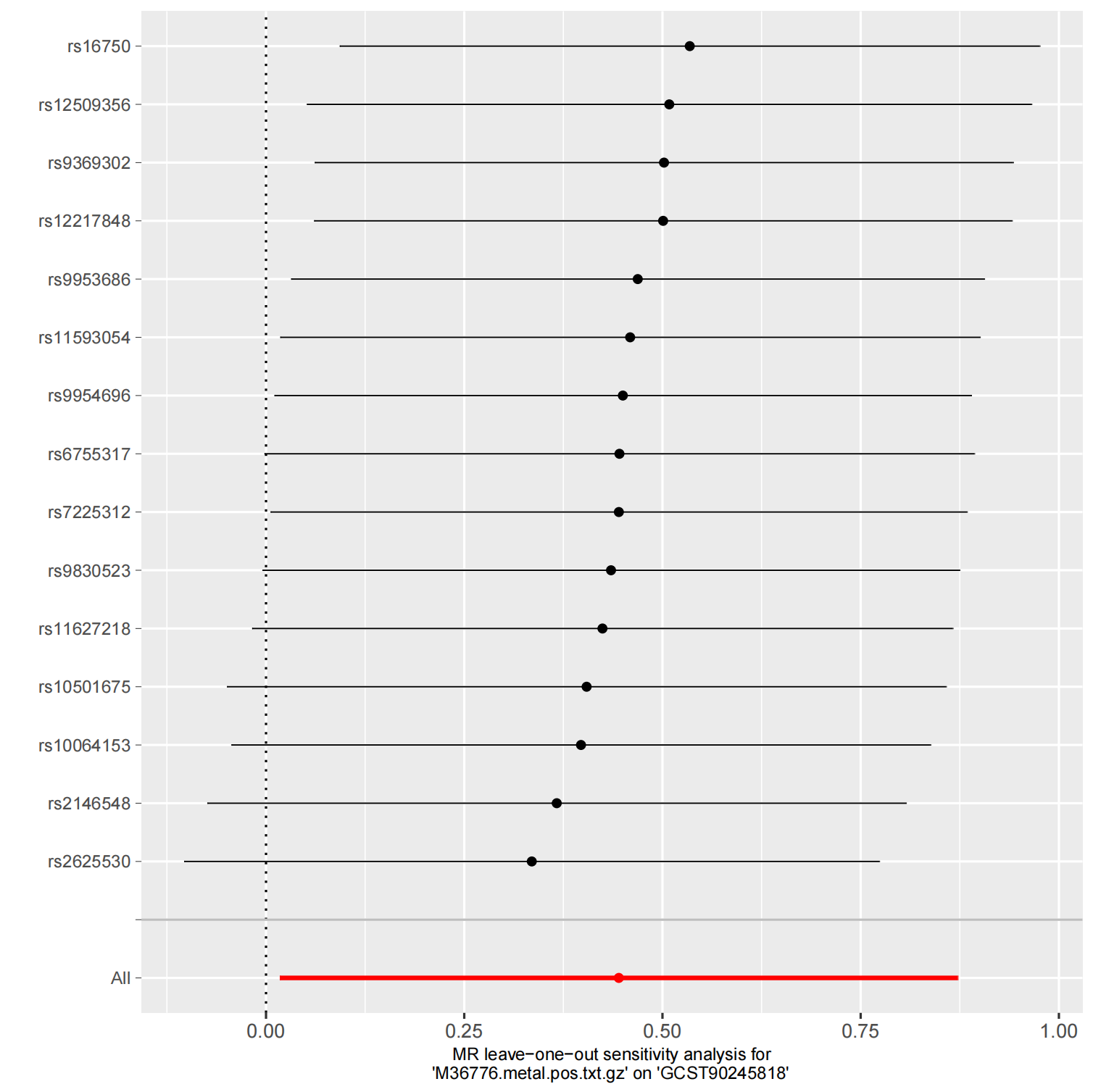

Supplement: Supplementary file 1 — Figure S1. Figure S2. [file JOCD-24-e16763-s001.docx]
